# Effects of E-Cigarette Flavoring Chemicals on Human Macrophages and Bronchial Epithelial Cells

**DOI:** 10.3390/ijerph182111107

**Published:** 2021-10-22

**Authors:** Anna M. Morris, Stephen S. Leonard, Jefferson R. Fowles, Theresa E. Boots, Anna Mnatsakanova, Kathleen R. Attfield

**Affiliations:** 1 Health Effects Laboratory Division, National Institute for Occupational Safety and Health, Morgantown, WV 26505, USA; annamanzi421@gmail.com (A.M.M.); sel5@cdc.gov (S.S.L.); oph6@cdc.gov (T.E.B.); fma8@cdc.gov (A.M.); 2 Department of Basic Pharmaceutical Sciences, West Virginia University Health Sciences Center, Morgantown, WV 26505, USA; 3 Environmental Health Investigations Branch, California Department of Public Health, Richmond, CA 94804, USA; jeff.fowles@cdph.ca.gov

**Keywords:** electronic cigarettes, e-cigarettes, flavorings, toxicity, airway epithelium, macrophages, inflammation

## Abstract

E-cigarettes utilize a wide range of flavoring chemicals with respiratory health effects that are not well understood. In this study, we used pulmonary-associated cell lines to assess the in vitro cytotoxic effects of 30 flavoring chemicals. Human bronchial epithelial cells (BEAS-2B) and both naïve and activated macrophages (THP-1) were treated with 10, 100, and 1000 µM of flavoring chemicals and analyzed for changes in viability, cell membrane damage, reactive oxygen species (ROS) production, and inflammatory cytokine release. Viability was unaffected for all chemicals at the 10 and 100 µM concentrations. At 1000 µM, the greatest reductions in viability were seen with decanal, hexanal, nonanal, cinnamaldehyde, eugenol, vanillin, alpha-pinene, and limonene. High amounts of ROS were elicited by vanillin, ethyl maltol, and the diketones (2,3-pentanedione, 2,3-heptanedione, and 2,3-hexanedione) from both cell lines. Naïve THP-1 cells produced significantly elevated levels of IL-1β, IL-8, and TNF-α when exposed to ethyl maltol and hexanal. Activated THP-1 cells released increased IL-1β and TNF-α when exposed to ethyl maltol, but many flavoring chemicals had an apparent suppressive effect on inflammatory cytokines released by activated macrophages, some with varying degrees of accompanying cytotoxicity. The diketones, L-carvone, and linalool suppressed cytokine release in the absence of cytotoxicity. These findings provide insight into lung cell cytotoxicity and inflammatory cytokine release in response to flavorings commonly used in e-cigarettes.

## 1. Introduction

Electronic nicotine delivery systems (ENDS), including e-cigarettes and other vaping devices, have gained popularity since their introduction to the U.S. market within the last decade [1,2,3]. They were initially marketed as potentially safer alternatives to tobacco cigarettes, but with many young adults and teens adopting ENDS use regardless of tobacco cigarette consumption [1], it is vital to understand the associated health impacts of all ingredients and device components [4,5,6]. These battery powered devices contain a chamber filled with e-liquid, which is heated by a metallic filament to produce an aerosol which the user then inhales. E-liquid is typically a mixture of propylene glycol (PG), vegetable glycerin (VG), nicotine ranging from 0 to 3.6% by volume [7], and a wide range of flavoring chemicals [8].

Many of these flavoring chemicals are considered to be “Generally Recognized As Safe” (GRAS) to ingest by the U.S. Food and Drug Administration (FDA), through evaluations by the Flavor Extracts Manufacturers Association [9]. Similarly, the World Health Organization Joint Expert Committee on Food Additives and Contaminants (JECFA) has carried out safety evaluations of food flavoring chemicals [10]. These evaluations, however, can mislead consumers’ understanding of the flavorings’ safety because these assessments do not ordinarily include an assessment of inhalation toxicity. Additionally, these assessments have not addressed a cumulative systemic dose that includes e-cigarette use, which could potentially create a much higher dose for moderate to heavy e-cigarette users.

A growing number of studies provide evidence that e-liquids and their aerosols can be cytotoxic and cause cellular indicators of respiratory irritation and inflammation [6,11,12,13,14,15,16]. Some flavoring chemicals also have established respiratory toxicity [17]. For instance, the commonly used butter flavoring diacetyl (2,3-butanedione) was found to cause severe inflammation and scarring of the bronchioles when inhaled, resulting in bronchiolitis obliterans in occupational settings [18]. A number of common flavoring chemicals, including diacetyl, cinnamaldehyde, acetoin, 2,3-pentanedione, vanillin, maltol and coumarin, induce oxidative stress and cytokine release [19,20]. Additionally, the flavoring chemicals dipentene, ethyl maltol, citral, linalool, and piperonal were found to generate free radicals when vaporized [21]. Many flavoring chemicals are aldehydes, which are frequently primary irritants of mucosal tissue of the respiratory tract [22]. More recently, certain flavoring chemicals have been found to inhibit innate immune responses, with cinnamaldehyde and ethyl vanillin decreasing neutrophil oxidative burst, and benzaldehyde impairing phagocytosis [23]. Disrupting the balance of inflammatory processes in the lung carries health implications. Chronic lung inflammation from repeated exposures can lead to tissue damage, scarring, asthma, and obstructive pulmonary disease, while suppression of normal immune cell actions may increase susceptibility to infection [24].

While flavoring chemicals are now receiving more research attention [17,19,20,21,22,23], only a limited number of flavoring chemicals that comprise the thousands of e-liquid flavors available on the market have been assessed for respiratory toxicity. Additionally, e-liquid formulations, both commercial and homemade, are composed of a myriad of flavoring chemical combinations which can change over time, in concentration, and between brands [25], all of which can complicate investigations of e-liquid toxicity. For example, a study of 277 products identified up to 50 flavoring chemicals per product, with total flavoring chemical concentrations up to 362 mg/mL (37% were above 10 mg/mL) [8]. Studies on flavoring chemicals in liquid form, while one step removed from exposures under use conditions, provide a basis for prioritizing chemical hazards and further assessing combinations or commercial mixtures of flavoring chemicals under conditions of actual use. To address these gaps in toxicity hazard data for flavoring chemicals, our study assessed effects of 30 individual flavoring chemicals on cell death, ROS production, and inflammatory cytokine markers in two cell lines associated with the lung: human bronchial epithelial cells (BEAS-2B) and human monocytes (THP-1).

## 2. Materials and Methods

### 2.1. Cell Culture

Human bronchial epithelial cells (BEAS-2B) were used due to their well established, wide use in toxicological studies of various pollutants and tobacco products [26]. THP-1 cells are also commonly used in toxicological studies, as they can be differentiated to model alveolar macrophages (AM), the primary immunological defense for the blood–air barrier [27,28]. All cells were cultured at 37 °C in a 5% CO_2_ atmosphere in a Thermo Forma incubator (Thermo Fisher, Waltham, MA, USA). BEAS-2B cells (ATCC, Manassas, VA, USA) were cultured in DMEM:F12 media (ATCC) supplemented with 10% fetal bovine serum and 50 mg/mL penicillin/streptomycin (ATCC). BEAS-2B cells were seeded at a density of 10,000 cells per well in a 96-well plate and grown to ~80% confluency for all experiments. THP-1 cells (ATCC) were cultured in RPMI-1640 supplemented with 10% fetal bovine serum (ATCC), 50 mg/mL penicillin/streptomycin, and 0.004% 2-mercaptoethanol (Sigma-Aldrich, St. Louis, MO, USA). The THP-1 monocytes were differentiated into naïve macrophages by stimulating them with 150 nM vitamin D3 (Sigma-Aldrich, St. Louis, MO, USA) for 48 h and 10 nM phorbol 12-myristate 13-acetate (PMA) for 12 h (Sigma-Aldrich). To differentiate the naïve macrophages (M0) into classically activated macrophages (M1), they were stimulated with 10 ng/mL of LPS (Sigma-Aldrich) [29]. THP-1 cells were seeded at a density of 20,000 cells per well in a 96-well plate for all experiments. Cell line authentication was performed by the Genomics Core Facility at West Virginia University, Morgantown, WV to confirm their identity.

### 2.2. E-Liquid Flavoring Chemical Selection

An initial master list of 89 flavoring chemicals was assembled from published literature or in-house e-liquid studies [30,31,32] and narrowed to 30 chemicals (Table 1) based on relative frequency of detection in literature of e-cigarette liquid chemical analyses, diversity of chemical structures, lack of inhalation toxicity data richness, and presence on the Flavor and Extract Manufacturers Association of the United States (FEMA) priority lists [32]. Exceptions regarding diversity were made for several of the buttery flavoring chemicals (diketones), which were included either due to their known associations with severe respiratory disease or their potential to serve as substitutes [18,33]. Additionally, where various versions of the same acid, aldehyde, or alcohol existed, we selected the aldehyde due to its higher reactivity and potential as a respiratory irritant [22]. Also taken into consideration was their consistency and completeness of availability for purchase through one vendor, Sigma-Aldrich, whether they were available in high purity and “food grade,” and whether their physical characteristics were appropriate for in vitro assays (i.e., not suspended in ethanol or methanol).

### 2.3. Preparation of E-Liquid Solutions

Due to low water solubility of several flavoring compounds, a vehicle solution of PG and VG (Glycerin Supplier, Houston, TX, USA) was used. A 1% solution of a 50 PG/50 VG by volume mixture was found to be sub-toxic, retained its solvent properties, and exhibited no significant difference from the phosphate buffer solution (PBS) control for all endpoints tested. Solutions (100 mM) of flavoring chemicals were prepared at room temperature in a 100% 50/50 solution and diluted in PBS to 1% PG/VG. Concentrations of both the liquid and solid flavoring chemicals were added to the PG/VG vehicle and vortexed until the solution was mixed at room temperature. The 100 mM flavoring chemical solutions were stored at 4 °C for the duration of the study and used within 48 h of preparation; however, the chemical stability of the flavorings in the dosing solvent was not tested. Cells were treated with flavoring chemicals using final concentrations of 10, 100, and 1000 µM diluted in PBS. This concentration range has been used in other published in vitro investigations of e-liquid flavoring chemical effects [17,18]. Our chosen concentration range of flavorings is expected to be relevant to in vivo exposures; however, an exposure assessment with modeling of lung deposition and absorption is not provided in this paper, but would be needed to establish in vivo exposures and dose thresholds. Flavoring chemicals within e-cigarette liquids themselves have been documented to vary widely, at upper ranges of hundreds of mM concentrations [8,19,22]. All flavoring chemicals were classified as “Food Grade” and obtained from Sigma-Aldrich.

### 2.4. Test for Interference

Assays for endotoxin, lactate dehydrogenase, intracellular reactive oxygen species (dichlorofluorescin diacetate [DCFH-DA]), and cytokines were all tested for assay interference through the use of controls, blanks, and E-liquid reaction tests.

### 2.5. Endotoxin

To determine whether any endotoxin was present in the flavoring chemical solutions, a Pierce™ Chromogenic Endotoxin Quantification assay (ThermoFisher, Waltham, MA, USA) was performed according to the manufacturer’s instructions and absorbance was read at 440 nanometer (nm). The 100 mM flavoring chemical solution was diluted 1:5 in endotoxin-free water.

### 2.6. Cellular Viability

Viability was assessed with the alamarBlue^TM^ assay (Thermo Scientific, Lenexa, KS, USA). alamarBlue^TM^ reports viability by reacting with FMNH_2_, FADH_2_, NADH, NADPH, and cytochromes to measure the entire reducing potential of the cell. Cells were treated with 10, 100, and 1000 µM of flavoring chemicals and incubated for 4 and 24 h. AlamarBlue (10 µL) was added to the medium in each well for a final volume of 100 µL and incubated for 4 h before reading. Fluorescence was measured at 560 ex/590 em with a Synergy H1 microplate reader (BioTek, Winooski, VT, USA). Values were compared to the 1% PG/VG vehicle control.

### 2.7. Lactate Dehydrogenase

Membrane damage was assessed with the Homogeneous Membrane Integrity Assay (Promega, Madison, WI, USA) according to the manufacturer’s instructions. Lactate dehydrogenase (LDH) is released from damaged cells into the culture medium. The assay utilizes a coupled enzymatic reaction which converts resazurin into resorufin. The fluorescent resorufin signal is directly proportional to the amount of LDH in the media. Cells were treated with 10, 100, and 1000 µM of the flavoring chemicals and incubated for 4 and 24 h, after which equal parts of media and reagent were mixed and incubated 10 min. Fluorescence was measured at 560 ex/590 em.

### 2.8. Intracellular ROS

Intracellular ROS were measured using the cell permeable dye, 2′,7′-DCFH-DA. DCFH-DA is de-esterified intracellularly and turns to highly fluorescent 2′,7′-dichlorofluorescein (DCF) upon oxidation. Cells were incubated with the dye for 45 min, after which the cells were rinsed with PBS and treated with 10, 100, and 1000 µM of the flavoring chemicals. The fluorescence intensity was measured at 480 ex/530 em on the microplate reader to quantify the amount of ROS produced by the cells after chemical exposure for 6 h. Separate wells of solely flavoring chemical and media were included in the plates and subtracted from their respective wells of treated cells to account for any autofluorescence.

### 2.9. Cytokines

Cells were grown in 96-well plates as previously indicated and treated with 1000 µM of flavoring chemical solution for 4 and 24 h. LPS (1 µg/mL) was used as a positive control. The medium was collected and frozen at −80 °C before assaying. BEAS-2B medium was undiluted for the assay, naïve THP-1 medium was diluted 1:10, and activated THP-1 medium was diluted 1:20 in the kit diluent. The cytokine analysis was conducted according to manufacturer’s instructions using the V-PLEX proinflammatory panel II (Meso Scale Diagnostics, Rockville, MD, USA), which quantifies IL-1β, IL-6, IL-8, and TNF-α.

### 2.10. Statistics

All experiments had three replicate wells and were repeated to give an n of 3. Significance was determined by comparing flavoring chemical treatments to the 1% PG/VG vehicle control. A one-way ANOVA coupled with Dunnett’s test for comparisons to the vehicle control was used to determine significance with SigmaPlot 14.0.

### 2.11. Supplemental Analysis

We explored machine learning methods of cluster analysis and random forest [34] algorithms to investigate predictions of the most important factors in overall assay outcomes based on their relative biological activity and toxicity (detailed methods in Supplemental Information). In short, we first established groupings of the 30 chemicals and the PG/VG vehicle control using hierarchical clustering. Data sets were enlarged with replicates (50, 100, and 150) for each chemical–assay–time point–cell type combination generated from normal distributions employing the corresponding sample mean and standard deviation. Physicochemical properties explored as predictors included molecular weight, density, solubility in H_2_O, safety data sheet hazard classifications, functional group list, and canonical simplified molecular-input line-entry system (SMILES). These were used in classification Random Forests to classify flavoring chemicals into previously established groups. All clustering and modeling analyses were conducted in R version 3.6.0 using the random Forest package for modeling [35].

## 3. Results

Initial assay assessment of the 10 and 100 µM concentrations indicated no effects on cell viability, LDH release, and ROS induction assays. The following sections report results from 1000 µM concentration experiments. In this preliminary set of studies, in order to screen and prioritize chemicals for future study, inflammatory cytokines (IL-1β, IL-6, IL-8 and TNF-α), were measured with the 1000 µM dose only. Summarized findings for all flavoring chemicals appear in Table 2. Detailed results for all chemicals are included in the Supplementary Figures.

### 3.1. Endotoxin Analysis

Endotoxin analysis showed negligible amounts present in all prepared concentrations and chemical additives. Measured amounts ranged from <0.055 to 0.074 EU, which were not statistically significant compared to endotoxin-free reagent water control included in kit (data not shown).

### 3.2. Cell Viability

**Figure 1:**
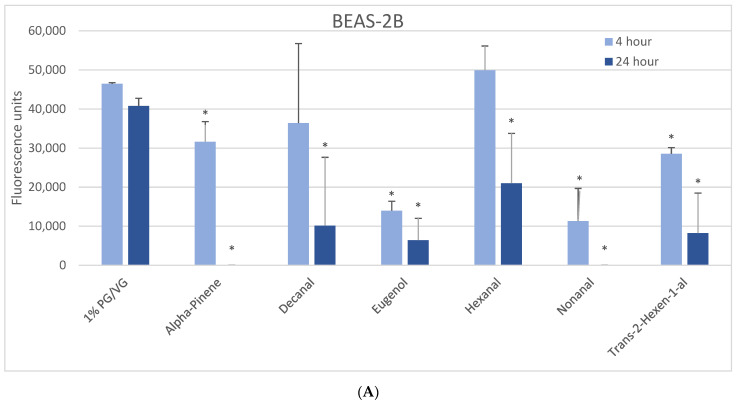

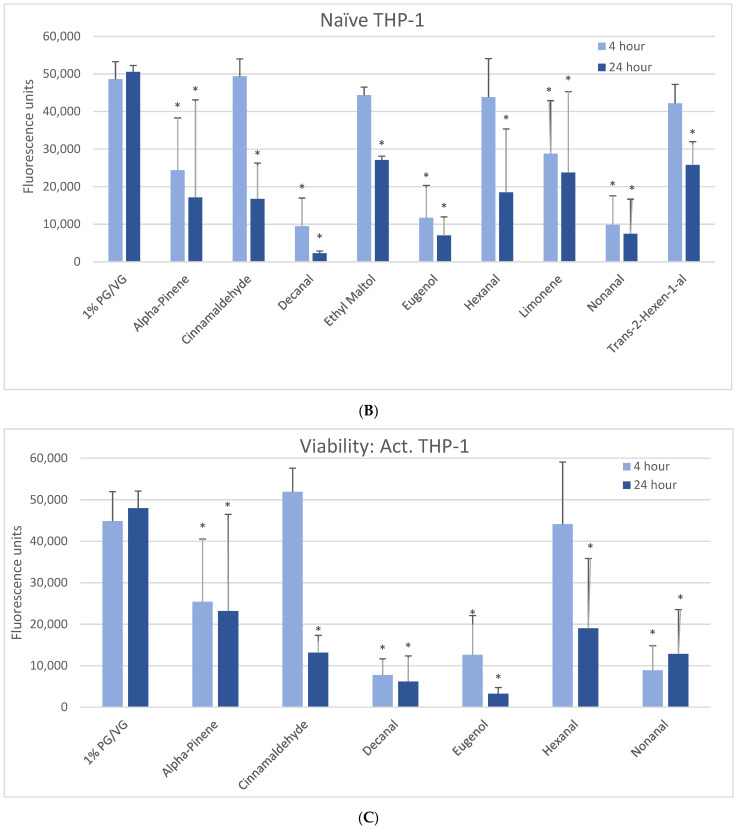
Viability effects of selected flavoring chemicals (1000 μM) after 4 and 24 h measured by alamarBlue fluorescence. (**A**) BEAS-2B cells, (**B**) naïve THP-1 cells, and (**C**) activated THP-1 cells. Data are presented as means ± standard error. Significance: * *p* < 0.05 as compared with vehicle control (1%PG/VG).

The 1000 µM exposure level showed some significant effects across cell types (Figure 1). A significant decrease in viability was observed in BEAS-2B cells after 4 h with eugenol, nonanal, and trans-2-hexen-1-al, with a lesser but still significant decrease with alpha-pinene (Figure 1A). After 24 h, a significant decrease in viability was observed with alpha-pinene, decanal, eugenol, hexanal, nonanal, and trans-2-hexen-1-al. A significant decrease in viability was observed in naïve THP-1 cells after 4 h in alpha-pinene, decanal, eugenol, limonene, and nonanal (Figure 1B) and after 24 h also seen with cinnamaldehyde, ethyl maltol, hexanal, and trans-2-hexen-1-al. Decanal, eugenol, and nonanal were the most cytotoxic at both timepoints. A significant decrease in viability was observed in the activated THP-1 cells after 4 h with alpha-pinene, decanal, eugenol, and nonanal (Figure 1C) and after 24 h also with cinnamaldehyde and hexanal. Differences in cell viability were not observed at either timepoint for twenty-one of the flavoring chemicals (Table 2, Supplemental Figure S1).

### 3.3. Lactate Dehydrogenase

The 1000 µM exposure level caused some significant effects across cell types (Table 2). In the BEAS-2B cells, alpha-pinene, decanal, and nonanal were the only flavoring chemicals to cause any level of significant LDH release at 4 h. However, the significance of this finding is uncertain since no significant effect was observed after 24 h. Naïve THP-1 cells elicited significant LDH release only at 24 h, with alpha-pinene, decanal, ethyl maltol, eugenol, hexanal, limonene, and nonanal. Similarly at 24 h, ethyl maltol, eugenol, and nonanal caused significant LDH release in activated THP-1 cells. THP-1 cells again appeared generally to be more sensitive than the BEAS-2B cells to the cytotoxic effects of the flavoring chemicals. Increases in levels of LDH release over control levels were not observed for thirteen flavoring chemicals (Table 2, Supplemental Figure S2).

### 3.4. Intracellular ROS

**Figure 2:**
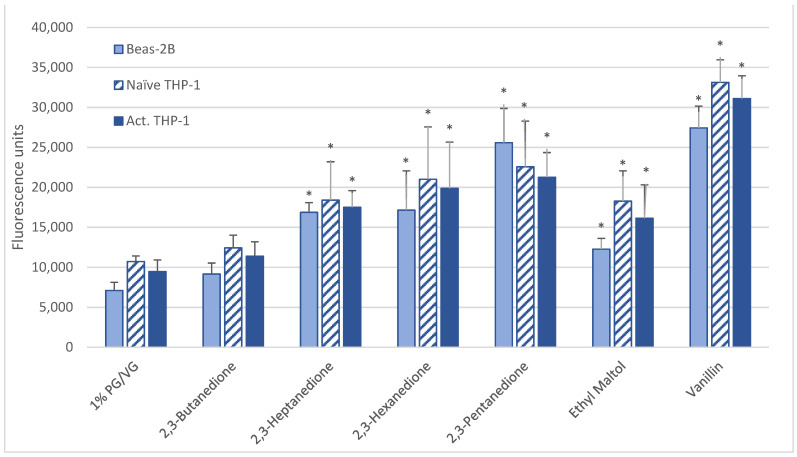
ROS measured with cell-permeable dye 2′,7′-dichlorofluorescin diacetate after 6-h treatment with 1000 µM of selected flavoring chemicals. Data are presented as means ± standard error. Significance: * *p* < 0.05 as compared with vehicle control (1%PG/VG).

After a 6-h 1000 µM exposure, all cell types produced significant ROS upon exposure to vanillin, ethyl maltol, and diketones (2,3-heptanedione, 2,3-hexanedione, 2,3-pentanedione) but not 2,3-butanedione (diacetyl) (Table 2, Figure 2). For 15 flavoring chemicals, ROS levels did not increase over those of the control (Table 2, Supplemental Figure S3).

### 3.5. Cytokines

Cytokine induction for both cell lines was measured, although cytokine production in BEAS-2B was minor, with levels for the only two inducing flavoring chemicals at levels 100- to 1000-fold lower than the THP-1 cell lines. Nonanal increased IL-1 β and IL-8 levels at the 4-h time point, and alpha-pinene increased IL-1β at the 4-h time point only in BEAS-2B cells (Table 2, Supplemental Figures S4A, S5A, S6A, and S7A). Detailed results for the more responsive THP-1 cell lines follow.

#### 3.5.1. IL-1β

**Figure 3:**
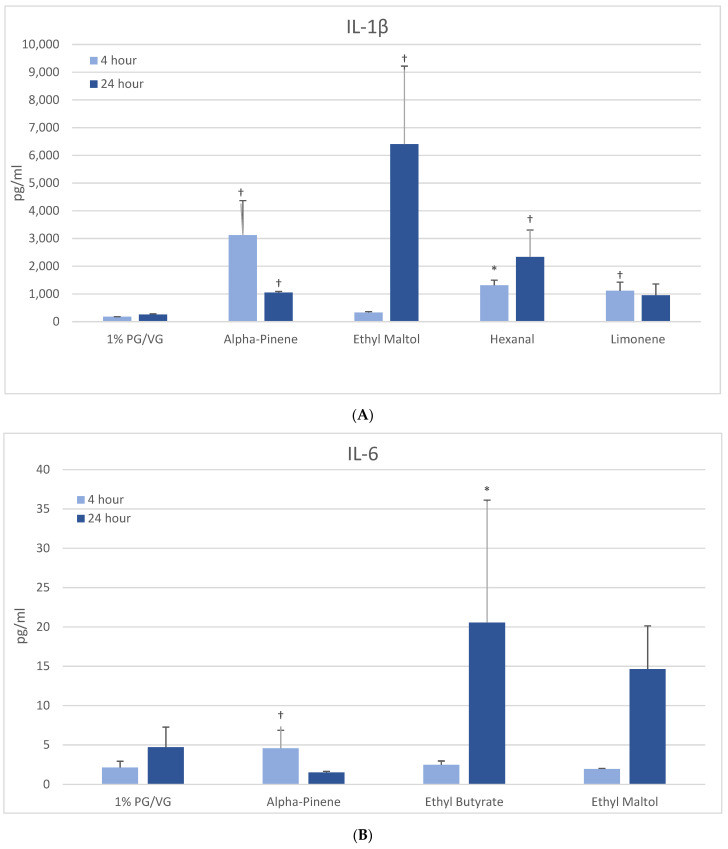

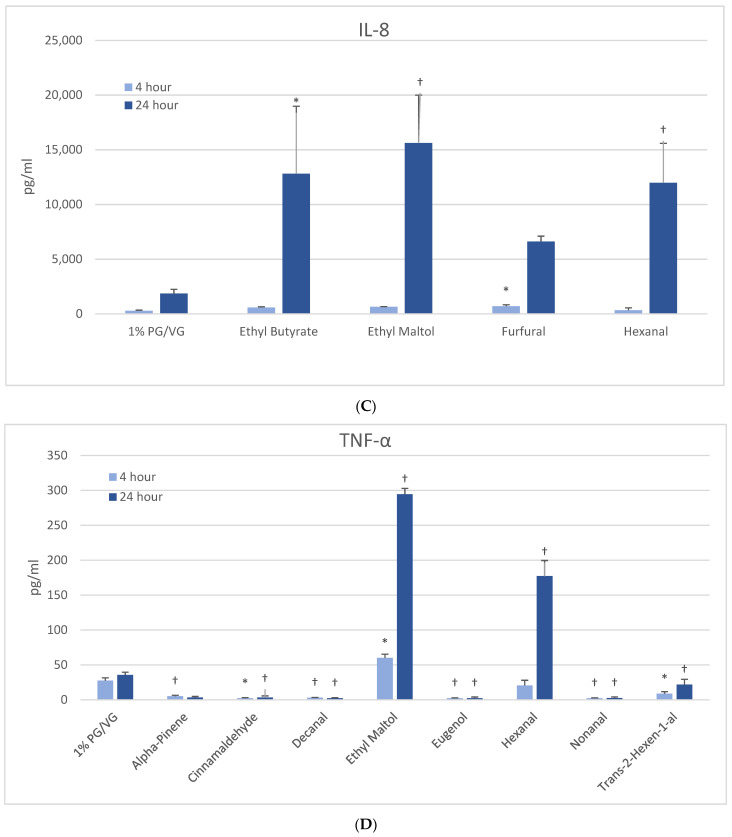
Cytokine production in naïve THP-1 cells measured in cell supernatants after 4- and 24-h treatment with 1000 µM of selected flavoring chemicals. (**A**) IL-1β; (**B**) IL-6; (**C**) IL-8; (**D**) TNF-α. Data are presented as means ± standard error. Significance: * *p* < 0.05 as compared with vehicle control (1%PG/VG) and ^†^ *p* < 0.05 with reduced viability (Figure 1). * *p* < 0.05, ^†^ *p* < 0.05 with reduced viability (Figure 1).

After 4 h, naïve THP-1 cells demonstrated significant increases in IL-1β after alpha-pinene, hexanal, and limonene treatment (Figure 3A) with further increases after 24 h observed with hexanal (9-fold) and ethyl maltol (25-fold). These chemicals had also decreased viability. In activated THP-1 cells, a significant increase in IL-1β was observed with alpha-pinene and ethyl maltol whose levels increased 5-fold over control at 24 h (Table 2, Supplemental Figure S4). Decreases in IL-1β were observed at both timepoints with exposure to cinnamaldehyde, decanal, eugenol, L-carvone, nonanal, and trans-2-hexen-1-al. Chemicals that had not impacted viability additionally were L-carvone, ethyl maltol, and trans-2-hexen-1-al. Twenty flavoring chemicals did not influence IL-1β concentrations significantly compared to controls.

#### 3.5.2. IL-6

**Figure 4:**
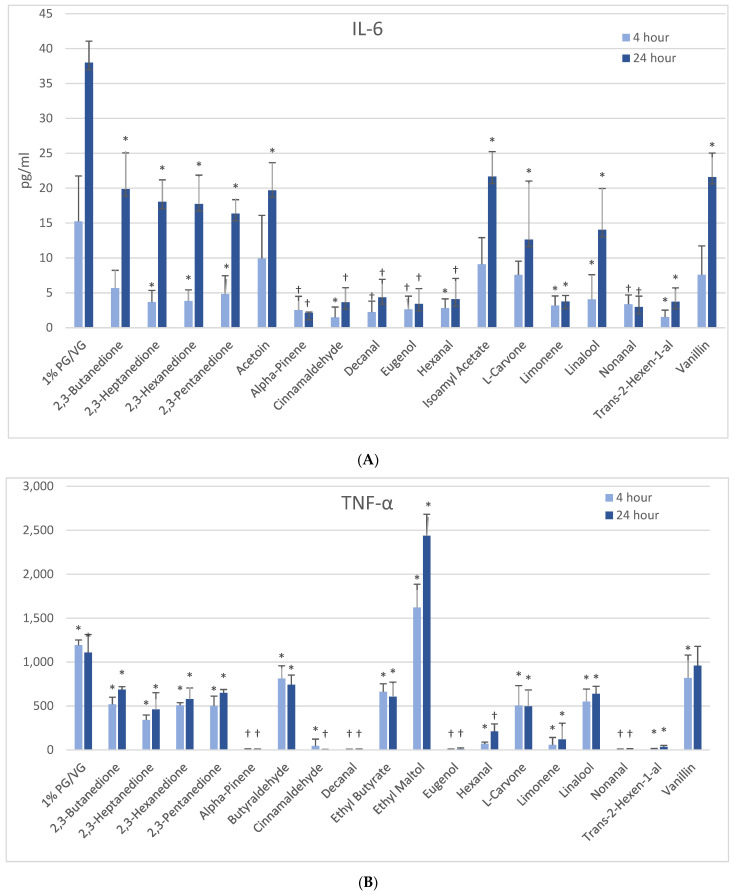
Cytokine production in activated THP-1 cells measured in cell supernatants after 4- and 24-h treatment with 1000 µM of selected flavoring chemicals. (**A**) IL-6; (**B**) TNF-α. Data are presented as means ± standard error. Significance: * *p* < 0.05 as compared with vehicle control (1%PG/VG) and ^†^ *p* < 0.05 with reduced viability (Figure 1).

At 4 h, naïve THP-1 cells exhibited modest elevation in IL-6 with one flavoring chemical (alpha-pinene) and more pronounced elevation with ethyl butyrate (4-fold) and ethyl maltol (3-fold) at 24 h (Figure 3B). Only alpha-pinene had attendant viability effects. Activated THP-1 cells demonstrated significant IL-6 suppression at 4 h with 12 flavoring chemicals (Figure 4A). After 24 h, the same 12 chemicals significantly suppressed IL-6 production by 52 to 94%; additionally, 2,3-butanedione, acetoin, isoamyl acetate, L-carvone, and vanillin also suppressed IL-6 production. Notably, cytokine suppression was unrelated to any cell viability decrease for the diketones, limonene, linalool, and L-carvone. Eleven flavoring chemicals did not affect IL-6 release at this concentration.

#### 3.5.3. IL-8

At 4 h, a modest but significantly increased production of IL-8 was noted only with furfural in naïve THP-1 cells (Figure 3C). After 24 h, three other flavoring chemicals additionally induced IL-8: ethyl butyrate (7-fold), ethyl maltol (8-fold) and hexanal (6-fold), with the latter two attendant to cell viability impacts. The activated THP-1 cells did not exhibit significantly increased IL-8 production compared to the vehicle control; however, eight flavoring chemicals significantly decreased IL-8 production by 47 to 99% (Table 2). Of these, trans-2-hexen-1-al and limonene treatments led to significant reductions in viability in the accompanying assays. Nineteen flavoring chemicals did not impact IL-8 release at this concentration.

#### 3.5.4. TNF-α

At 4 h, naïve THP-1 cells responded with a significant increase in TNF-α from ethyl maltol treatment (2-fold), whereas cinnamaldehyde and trans-2-hexen-1-al had suppressive effects (Figure 3D). Alpha-pinene, decanal, eugenol, and nonanal had suppressive effects additionally but in the context of reduced cell viability. At 24 h, only ethyl maltol and hexanal treated cells produced significant TNF-α elevations with 8- and 5-fold increases, respectively, despite cell viability impacts. Activated THP-1 cells showed far more significant effects from the flavoring chemicals than the naïve form with ethyl maltol being the only flavoring chemical to significantly increase TNF-α (2-fold). Suppression, without attendant cell viability (33% to 99% decrease), was observed at 4 and 24 h with 12 chemicals (Figure 4B). Twelve flavoring chemicals did not elicit THP-1 responses at this concentration.

### 3.6. Modeling

The modeling results indicated that the biologically active flavoring chemicals fall into the categories of aldehydes with large carbon chains attached, compounds containing aromatic rings, and chemicals classified as monoterpenes, which contain two isoprene units (Supplementary Figures S8–S10). Empirical toxicity profiles were best predicted by assessing the canonical SMILE signature for effects on BEAS-2B cells and functional group for THP-1 cells (Supplementary Figure S11).

## 4. Discussion

This study investigated toxicity through a series of tests on 30 flavoring chemicals using respiratory-associated cell lines. We observed that, at the highest concentration tested, 1000 µM, cell viability was affected across all three cell types at the 4-h time point by three flavoring chemicals (alpha-pinene, eugenol, and nonanal). Additionally, three other flavoring chemicals were active in at least one endpoint and cell type at this time point (decanal, limonene, and trans-2-hexen-1-al). Longer exposure to cinnamaldehyde, ethyl maltol, and hexanal led to viability impacts or cell membrane disruption, primarily in THP-1 cell lines. Twenty-one flavoring chemicals did not influence either viability or cell membrane disruption at these concentrations.

The pro-oxidants of note in our study were vanillin, ethyl maltol, 2,3-pentanedione, 2,3-hexanedione, and 2,3-heptanedione, though, unexpectedly, 2,3-butanedione did not behave similarly to the other diketones. Other studies have found the potential of e-liquids to induce reactive oxygen species in multiple cell types [16,36]. The consequences of this oxidative stress can result in apoptosis and sustained inflammation, which are implicated in a number of pulmonary diseases including asthma, fibrosis, chronic obstructive pulmonary disease, and lung cancer [36]. ROS overproduction can also be a consequence of peroxisome dysfunction, which normally regulates ROS generation and quenching within the cell [37].

Increased cytokine release was most markedly observed with ethyl maltol, the only flavoring chemical to produce solely increased inflammatory cytokine release (IL-1β, IL-6, and TNF-α). Alpha-pinene was the most potent inducer of IL-1β at 4 h, while hexanal induced increases in IL-1β, IL-8, and TNF-α in naïve THP-1 cells. Conversely, broad and profoundly suppressive activity of the inflammatory cytokines was found in activated THP-1 cells with many of the flavoring chemicals. Though some of these inflammatory cytokine decreases may be attributed to increased cell death, no change in cell viability in naïve or active macrophages was observed with the diketones, L-carvone, and linalool. With the activated THP-1 cells, several of the flavoring chemicals that are components of various essential oils had a suppressive effect on inflammation and inflammatory cytokine secretion, which is consistent with published assessments [38,39,40].

The assortment of patterns seen in the toxicity assays employed in this paper highlights the need for examining multiple endpoints to observe effects from flavoring toxicity assays. Looking across assays, while all chemicals with viability and LDH activity also displayed cytokine activity, several flavoring chemicals only demonstrated altered cytokine activity but not other activities: acetoin (IL-6), butyraldehyde (TNF-alpha), ethyl butyrate (IL-6, IL-8, TNF-α), and isoamyl acetate (IL-6). Nine flavoring chemicals in all (2,3,5-trimethylpyrazine, 2-acetylpyrazine, acetaldehyde, benzyl alcohol, menthol, ethyl acetate, isopropyl myristate, methyl salicylate) had no observable effects at 1000 µM concentrations.

Cinnamaldehyde is one of the flavoring chemicals that has received considerable study in relation to in vitro toxicity, immunotoxicity, and inflammatory cytokines [41,42,43]. Among the findings of previous studies on cinnamaldehyde or cinnamon e-cigarette flavorings is cytotoxicity to human lung epithelial cells along with a suppression of inflammatory cytokines and inhibition of non-specific immune responses of macrophages, neutrophils, and natural killer cells [16,41]. Our study found similar results, with cinnamaldehyde (1 mM) producing cytotoxicity to THP-1 but not BEAS-2B cells, and suppressing IL-1 β, IL-6, IL-8, and TNF-α in activated THP-1 cells.

Ethyl maltol has received recent attention in e-cigarette flavoring toxicity studies [44], including evidence of cytotoxicity after heating and aerosolization [45]. Ethyl maltol has exhibited increased free radical creation over PG/VG alone (21) and also caused oxidative stress in lung epithelial cells in the presence of copper, through a proposed mechanism involving increased bioavailability of the metal ion and intracellular redox cycling [45]. Durrani and colleagues suggested that other metals relevant to e-cigarette components could follow a similar pattern of toxicity and noted that the combination of the metal and ethyl maltol increased cytotoxicity in a synergistic manner [45]. Our study similarly showed increased ROS production in all cell types with ethyl maltol treatment. The marked induction of IL-1β and TNF-α pro-inflammatory cytokines in naïve and activated macrophages has not yet been observed in other studies and adds to the cytotoxicity and oxidative stress findings reported by Durrani and colleagues [45]. Some parallels with the chemically-related maltol have been reported for ROS, viability, and IL-8 production in monocytes at similar concentrations [20]. It is unknown if a common mechanism exists between the two compounds. However, their common occurrence in e-liquids (32% of 16,839 e-liquids for ethyl maltol and 22.8% for maltol [22]) suggests that any pro-inflammatory effect in the lung and associated health effects of these chemicals should be prioritized for further characterization.

The effects of vanillin on cytotoxicity and immune cytokine release in vitro have been found to depend on the cell type in addition to its dose. One study found that vanillin did not cause cytotoxicity after 24 h in one human monocyte cell line (MM6 cells) at concentrations of up to 1 mM, but did cause cytotoxicity in another cell line (U937 cells) at 0.5 mM [20]. Vanillin also increased ROS at a concentration of 1 mM, similar to what we observed in our study. However, the cells used in their study also responded to vanillin with induced IL-6, whereas we found IL-6 to be suppressed in the activated THP-1 cells in our study. These authors did not use activated macrophages in their test system. Vanillin and ethyl maltol were found to produce ROS in cell-free systems [20]. A study on hPF and A549 cells found vanillin to be cytotoxic in vitro following aerosolization [43]. Smith and colleagues found vanillin to perturb anti-oxidant pathways relevant to human disease in BEAS2B cells [46]. The ability of vanillin to exhibit anti-oxidant properties is likely due to the radical-scavenging activity via the homolytic fragmentation of the O-H bond [47]. Like ethyl maltol, vanillin’s frequent use as flavoring chemical (35% of 16,839 e-liquids [22]) raises concerns for a full understanding of its potential for respiratory harm.

dl-Menthol is a common additive in menthol cigarettes and has become an additive in e-liquids as well. A prior study found dl-menthol to suppress interferon-γ and downregulate Th1 lymphocyte activity in human peripheral blood mononuclear cells slightly at a concentration of 0.319 mM and strongly at a concentration of 5.1 mM [48], in the absence of cytotoxicity. Rickard and colleagues found no cytotoxicity of menthol in liver HepG2 cells exposed to 2.5 mM for 48 h [44]. We similarly did not observe significant cytotoxicity from dl-menthol and the differing anti-inflammatory effects seen in our study might be attributed to the 5-fold higher concentrations used by Bayat and colleagues [48].

Butter flavoring chemicals of the alpha-dicarbonyl family are frequently detected in e-liquids and have been indicated in the severe lung disease bronchiolitis obliterans and airway epithelial cytotoxicity. Consistent with prior studies, our study found that viability of bronchial epithelial cell lines were not affected at tested concentrations. However, other monocyte cell lines have indicated viability declines with acetoin, diacetyl, and 2,3-pentanedione at higher concentrations [16]. Given the reactive nature of the dicarbonyl structure, reactive oxygen species production is expected and ROS and RNA transcript markers of ROS induction have been observed previously [16,49] and also in our study, though not at significant levels with diacetyl. Reported inductions of inflammatory cytokines from diacetyl have predominantly been in the form of IL-8 increases [19,20], while our results exhibited more evidence of cytokine decreases (IL-6 and TNF-a). Results from both in vitro and in vivo toxicity studies indicate that the 2,3-pentanedione and longer diketones are not safe replacements for diacetyl [33]. Additionally, the substitute acetoin has been observed to convert to diacetyl in e-liquids over storage periods [50]. Concerningly, inhibition of diacetyl breakdown by other flavoring chemicals has been observed [51], potentially altering clearance kinetics and leading to additional concerns of flavoring chemicals mixtures within a single product.

In looking for patterns within the different assay types and physicochemical properties of the flavoring chemicals (solubility, molecular weight, density, functional groups, and canonical SMILES), our data set suggested that the most effective way to accurately predict flavoring chemical toxicity relative to the study’s battery of tests is by assessing the canonical SMILE signature for effects on BEAS-2B cells and functional group for THP-1 cells. The modeling results indicated that the biologically active flavoring chemicals fall into the categories of aldehydes with large carbon chains attached, compounds containing aromatic rings, and chemicals classified as monoterpenes, which contain two isoprene units.

One limitation of our study is that we examined the intrinsic cytotoxicity and cytokine production effects of individual flavoring chemicals, but not the effects after heating and aerosolization. However, studies on flavoring chemicals in liquid form, measured comparatively in the same system, provide a basis for prioritizing and further assessing combinations or commercial mixtures of chemicals under conditions of actual use. While only one dose was used for cytokine production, further sub-cytotoxic dose evaluations of cytokine effects are planned on the e-liquid flavorings of greatest interest. The mechanisms of induction or suppression of pro-inflammatory cytokines of individual chemicals require further investigation to better understand and predict pulmonary effects of these chemicals in their commercial mixture formulations. Potential future in vitro assays on flavoring chemicals could include assessment of DNA damage, mitochondrial integrity, glutathione and superoxide dismutase (SOD) activity, NF-ĸB and caspase-1 activation, as well as apoptosis and necrosis. Our study is intended to provide information on the inherent properties and hazards of the studied chemicals situated within a market of far ranging flavoring chemical concentrations. Therefore, future studies on a smaller list of chemicals, with a narrower concentration range, combined with lung tissue dose modeling, will be required to estimate threshold levels for risk assessment purposes.

Public attention to health concerns about e-cigarette use skyrocketed when deaths and hospitalizations occurred during the e-cigarette, or vaping, product associated lung injury (EVALI) outbreak of the latter half of 2019 through early 2020 [52]. While this outbreak has ultimately been linked to a diluting agent (vitamin E acetate) in illicit THC e-liquids and potential thermal breakdown products [16,53], the attention to these devices has also highlighted the existing, but under recognized, health concerns, of increased incidence of respiratory disease, such as asthma and COPD [6,54], as well as findings of inflammatory responses in the lungs and respiratory-related cell types [6,17,55]. Though the novelty of e-cigarettes has not yet allowed sufficient time for a full determination of their long-term impact, these short-term studies support concerns about the chronic health impacts, particularly in light of the enormous increase in flavored e-cigarette uptake in youth [1,2].

## 5. Conclusions

In conclusion, our study of 30 e-cigarette flavoring chemicals in a 1000 μM concentration found variable and complex effects on cytotoxicity and inflammatory cytokine production in human BEAS-2B lung epithelial cells and THP-1 pulmonary macrophages. Cytotoxicity was not observed with any chemical at 10 and 100 uM. The most cytotoxic chemicals across both cell lines were: alpha-pinene, decanal, eugenol, hexanal, nonanal, and trans-2-hexen-1-al. THP-1 cells were additionally sensitive to cytotoxicity from cinnamaldehyde, ethyl maltol, and limonene. Both pro- and anti-inflammatory effects were observed, depending on the chemical, with ethyl maltol the most pro-inflammatory flavoring, inducing IL-1β, IL-8, and TNF-α, while also causing cytotoxicity. Reactive oxygen species were significantly induced by vanillin, ethyl maltol, and the diketone class, including 2,3-pentanedione, 2,3-hexanedione, and 2,3-heptanedione, but not by 2,3-butanedione. Modeling suggested that canonical SMILES is a useful way to group and explore structural activity considerations. Further research should address the dosimetry of these chemicals in vivo, the various mechanisms of toxicity and the consequences of long-term use of these complex mixtures. These findings provide insight into the potential for lung cytotoxicity, inflammation, and oxidative stress that is presented by the use of e-cigarettes and provide a basis for future experiments with flavoring chemical e-liquid or aerosol exposures. Collectively, our findings suggest that flavored e-cigarette users could be at risk for inhalation toxicity across a range of flavoring types and further study of the risk for perturbance of normal pulmonary immune and inflammatory processes is in order.

## Figures and Tables

**Table 1:** List of flavoring chemicals and their characteristics.

E-liquid Flavoring Chemical	Flavor Profile	H_2_O Solubility (mM) *	Chemical Class	Molecular Structure
2,3,5-Trimethylpyrazine	Cocoa; Nutty	2.6 × 10^2^	Methyl, pyrazine	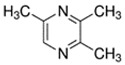
2,3-Butanedione	Buttery; Sweet	4.0 × 10^3^	acetyl, ketone	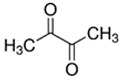
2,3-Heptanedione	Buttery; Dairy	2.6 × 10^2^	acetyl, ketone	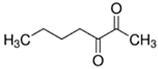
2,3-Hexanedione	Creamy; Fruity	8.7 × 10^2^	acetyl, ketone	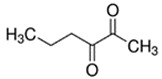
2,3-Pentanedione	Buttery; Caramel	2.8 × 10^3^	acetyl, ketone	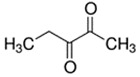
2-Acetylpyrazine	Bready; Nutty	8.9 × 10^3^	acetyl, pyrazine	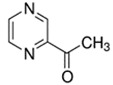
Acetaldehyde	Tart; Green Apple	2.3 × 10^4^	acetyl, aldehyde	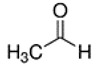
Acetoin	Creamy; Dairy	1.1 × 10^4^	acetyl, hydroxyl	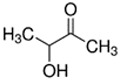
Alpha-pinene [(−)-α-pinene]	Cedarwood, Pine	1.8 × 10^−4^	isoprene, methyl	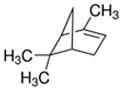
Benzyl Alcohol	Cherry; Almond	2.1 × 10^3^	benzene, hydroxyl	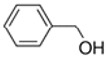
Butyraldehyde	Cocoa; Green	9.8 × 10^2^	aldehyde	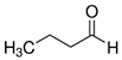
Cinnamaldehyde	Cinnamon; Spice	1.1 × 10^0^	benzene, aldehyde	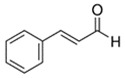
Decanal	Citrus; Orange peel	8.7 × 10^−2^	aldehyde	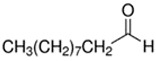
DL-Menthol	Menthol; Minty	2.9 × 10^0^	isoprene, hydroxyl	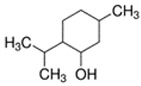
Ethyl Acetate	Fruity; Grape	9.1 × 10^2^	ester	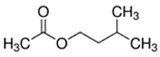
Ethyl Butyrate	Fruity; Apple	5.3 × 10^1^	ester	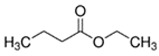
Ethyl Maltol	Sweet; Sugary	2.1 × 10^2^	hydroxyl, pyrone	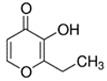
Eugenol	Clove; Spice	2.7 × 10^1^	hydroxyl, methoxy, benzene	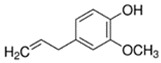
Furfural	Bready; Nutty	8.0 × 10^2^	aldehyde, furan	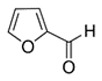
Hexanal	Fruity; Green	5.0 × 10^1^	aldehyde	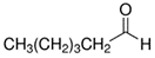
Isoamyl Acetate	Fruity; Banana	1.4 × 10^1^	ester	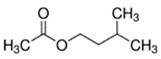
Isopropyl Myristate	Cheesy; Dairy	Insoluble (5.1 × 10^−5^)	ester	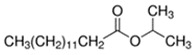
L-Carvone	Spearmint; Caraway	8.7 × 10^0^	isoprene, ketone	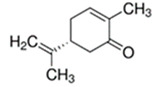
Limonene [(R)-(+)-Limonene]	Citrus; Orange	1.0 × 10^−1^	isoprene, methyl	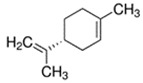
Linalool	Lemon; Lavender	1.0 × 10^1^	isoprene, hydroxyl	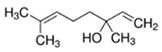
Methyl Salicylate	Wintergreen; Peppermint	2.7 × 10^1^	ester, hydroxyl, benzene	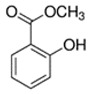
Nonanal	Green; Lemon	6.8 × 10^−1^	aldehyde	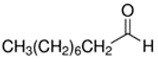
Propionaldehyde	Floral; Grape	4.5 × 10^3^	aldehyde	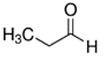
Trans-2-Hexen-1-al	Fruity; Green	7.5 × 10^1^	aldehyde	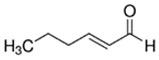
Vanillin	Sweet; Vanilla	7.2 × 10^1^	aldehyde, benzene, hydroxyl, methoxy	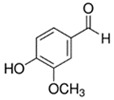

* Source: USEPA: https://comptox.epa.gov/dashboard/dsstoxdb/results?search=DTXSID1047075#properties (accessed on 23 September 2021). All values are median experimental values where available, otherwise median or average predicted values.

**Table 2:** Results summary expressing magnitude of change compared to vehicle control. B = BEAS-2B, N = Naïve THP-1, A = Activated THP-1. Arrows indicate significant difference (*p* < 0.05) to control: low decrease (small down arrow), twofold or greater decrease (large down arrow), low increase (small up arrow), twofold or greater increase (large up arrow).

Flavoring	4 h																		6 h			24 h																	
Chemical	Viability			LDH			IL-1β			IL-6			IL-8			TNF-α			ROS			Viability			LDH			IL-1β			IL-6			IL-8			TNF-α		
	B	N	A	B	N	A	B	N	A	B	N	A	B	N	A	B	N	A	B	N	A	B	N	A	B	N	A	B	N	A	B	N	A	B	N	A	B	N	A
2,3,5-Trimethylpyrazine																																							
2,3-Butanedione																		**↓**															🠓						🠓
2,3-Heptanedione												**↓**						**↓**	**↑**	🠑	🠑												**↓**						**↓**
2,3-Hexanedione												**↓**						**↓**	**↑**	**↑**	**↑**												**↓**						**↓**
2,3-Pentanedione												**↓**						**↓**	**↑**	**↑**	**↑**												**↓**						🠓
2-Acetylpyrazine																																							
Acetaldehyde																																							
Acetoin																																	🠓						
Alpha-Pinene	🠓	🠓	🠓	**↑**			**↑**	**↑**	🠑		**↑**	**↓**			**↓**		**↓**	**↓**				**↓**	**↓**	**↓**		**↑**							**↓**			**↓**			**↓**
Benzyl alcohol																																							
Butyraldehyde																		🠓																					🠓
Cinnamaldehyde									**↓**			**↓**			**↓**		**↓**	**↓**					**↓**	**↓**						**↓**			**↓**			**↓**			**↓**
Decanal		**↓**	**↓**	**↑**					**↓**			**↓**			**↓**		**↓**	**↓**				**↓**	**↓**	**↓**		**↑**				**↓**			**↓**			**↓**			**↓**
DL-Menthol																																							
Ethyl Acetate																																							
Ethyl Butyrate																		🠓														**↑**			**↑**				🠓
Ethyl Maltol									🠑								**↑**	🠑	🠑	🠑	🠑		🠓			**↑**	**↑**		**↑**	**↑**					**↑**			**↑**	**↑**
Eugenol	**↓**	**↓**	**↓**						**↓**			**↓**			**↓**		**↓**	**↓**				**↓**	**↓**	**↓**		**↑**	🠑			**↓**			**↓**			**↓**			**↓**
Furfural														🠑																									
Hexanal								**↑**				**↓**			**↓**			**↓**				🠓	**↓**	**↓**		**↑**			**↑**				**↓**		**↑**			**↑**	**↓**
Isoamyl Acetate																																	🠓						
Isopropyl Myristate																																							
L-Carvone									**↓**									**↓**												**↓**			**↓**						**↓**
Limonene		🠓						**↑**				**↓**			**↓**			**↓**					**↓**			**↑**							**↓**			**↓**			**↓**
Linalool												**↓**						**↓**															**↓**						🠓
Methyl Salicylate																																							
Nonanal	**↓**	**↓**	**↓**	**↑**			**↑**		**↓**			**↓**			**↓**		**↓**	**↓**				**↓**	**↓**	**↓**		**↑**	**↑**			**↓**			**↓**			**↓**			**↓**
Propionaldehyde																																							
Trans-2-Hexen-1-al	🠓								🠓			**↓**			**↓**		**↓**	**↓**				**↓**	🠓							**↓**			**↓**			**↓**			**↓**
Vanillin																		🠓	**↑**	**↑**	**↑**												🠓						

## Data Availability

Data are available from NIOSH upon request to the corresponding author.

## Supplementary Materials

The following are available online at https://www.mdpi.com/article/10.3390/ijerph182111107/s1, Supplementary Analysis—Modeling of predictors of toxicity profiles. Detailed Methods and Results. Supplementary Figures key—ELCF—Electronic Liquid Flavoring Chemical. Figure S1. (A) Viability of BEAS-2B epithelial cells after 4- and 24-h treatment with 1000 µM of flavoring chemicals measured by alamarBlue fluorescence. (B) Viability of naïve THP-1 cells after 4- and 24-h. (C) Viability of activated THP-1 cells after 4- and 24-h. Significant *p*-values are displayed in graph. Figure S2. (A) Membrane damage was assessed by lactate dehydrogenase (LDH) release after 4- and 24-h treatment with 1000 µM of flavoring chemicals in BEAS-2B cells, (B) naïve THP-1 cells, and (C) activated THP-1 cells. Figure S3. (A) ROS were measured with cell-permeable dye 2′,7′-Dichlorofluorescin diacetate and read after 6-h treatment with 1000 µM of flavoring chemicals for BEAS-2B cells, (B) naïve THP-1 cells, and (C) the activated THP-1 cells. Figure S4. (A) IL-1β in cell supernatants after 4- and 24-h treatment with 1000 µM of flavoring chemicals for BEAS-2B cells, (B) naïve THP-1 cells, and (C) activated THP-1 cells. * Indicates no significant decrease in viability accompanying decrease in cytokine production. Figure S5. (A) IL-6 in cell supernatants after 4- and 24-h treatment with 1000 µM of flavoring chemicals for BEAS-2B cells, (B) naïve THP-1 cells, and (C) activated THP-1 cells. * Indicates no significant decrease in viability accompanying decrease in cytokine production. Figure S6. (A) IL-8 in cell supernatants after 4- and 24-h treatment with 1000 µM of flavoring chemicals for BEAS-2B cells, (B) naïve THP-1 cells, and (C) activated THP-1 cells. * Indicates no significant decrease in viability accompanying decrease in cytokine production. Figure S7. (A) TNF-α in cell supernatants after 4- and 24-h treatment with 1000 µM of flavoring chemicals for BEAS-2B cells, (B) naïve THP-1 cells, and (C) activated THP-1 cells. * Indicates no significant decrease in viability accompanying decrease in cytokine production. Figure S8. Heat map of clustering of toxicity tests in BEAS-2B. (A) 4-h (B) 24-h. Figure S9. Heat map of clustering of toxicity tests in naïve THP-1. (A) 4-h (B) 24-h. Figure S10. Heat map of clustering of toxicity tests in activated THP-1. (A) 4-h (B) 24-h. Figure S11. Important physicochemical properties in accurately predicting a compound’s toxicity [34,35,56,57].
