# Supplementary material for: Effects of E-Cigarette Flavoring Chemicals on Human Macrophages and Bronchial Epithelial Cells
Source: Int J Environ Res Public Health. 2021 Oct 22;18(21):11107. doi: 10.3390/ijerph182111107 (PMC8583527; doi:10.3390/ijerph182111107)
Supplement: Supplementary file 1 [file ijerph-18-11107-s001.zip › ijerph-1392836-supplementary.pdf]

# Effects of E-Cigarette flavoring chemicals on Human Macrophages and Bronchial Epithelial Cells

Anna M. Morris[1,2], Stephen S. Leonard[1,2], Jeff R. Fowles[3], Theresa E. Boots[1], Anna Mnatsakanova[1], Kathleen R. Attfield[3,*]

[1]Health Effects Laboratory Division, National Institute for Occupational Safety and Health, Morgantown, WV

[2]West Virginia University Health Sciences Center, Morgantown, WV

[3]Environmental Health Investigations Branch, California Department of Public Health, Richmond, CA

*Address for correspondence: Kathleen R. Attfield, ScD

Environmental Health Investigations Branch, CDPH

850 Marina Bay Parkway, Building P

Richmond, CA 94804

**Supplementary Information**
**Table of Contents**


Supplementary Analysis – Modeling of predictors of toxicity profiles. Detailed Methods and Results.

Supplementary Figures key – ELCF - Electronic Liquid Flavoring Chemical

Figure S1. A) Viability of BEAS-2B epithelial cells after 4 and 24 h measured by alamarBlue fluorescence. B) Viability of naïve THP-1 cells after 4 and 24 hours. C) Viability of activated THP-1 cells after 4 and 24 hours. Significant p-values are displayed in graph.

Figure S2. A) Membrane damage was assessed by lactate dehydrogenase (LDH) release at 4 and 24 h in BEAS-2B cells, B) naïve THP-1 cells, and C) activated THP-1 cells.

Figure S3. A) ROS were measured with cell-permeable dye 2′,7′-Dichlorofluorescin diacetate and read at 6 h for BEAS-2B cells, B) naïve THP-1 cells, and C) the activated THP-1 cells.

Figure S4. A) IL-1β in cell supernatants after 4 and 24 h for BEAS-2B cells, B) naïve THP-1 cells, and C) activated THP-1 cells. *Indicates no significant decrease in viability accompanying decrease in cytokine production.

Figure S5. A) IL-6 in cell supernatants after 4 and 24 h for BEAS-2B cells, B) naïve THP-1 cells, and C) activated THP-1 cells. *Indicates no significant decrease in viability accompanying decrease in cytokine production.

Figure S6. A) IL-8 in cell supernatants after 4 and 24 h for BEAS-2B cells, B) naïve THP-1 cells, and C) activated THP-1 cells. *Indicates no significant decrease in viability accompanying decrease in cytokine production.

Figure S7. A) TNF-$\alpha$ in cell supernatants after 4 and 24 h for BEAS-2B cells, B) naïve THP-1 cells, and C) activated THP-1 cells. *Indicates no significant decrease in viability accompanying decrease in cytokine production.

Figure S8. Heat map of clustering of toxicity tests in BEAS-2B. A) 4 hour B) 24 hour

Figure S9. Heat map of clustering of toxicity tests in naïve THP-1. A) 4 hour B) 24 hour

Figure S10. Heat map of clustering of toxicity tests in activated THP-1. A) 4 hour B) 24 hour

Figure S11. Important physicochemical properties in accurately predicting a compound's toxicity.

Supplementary analysis - Modeling of predictors of toxicity profiles. Detailed Methods and Results.

Background

Due to the vast number of e-cigarette flavoring chemicals available on the market, we used machine learning to develop a method to illustrate an approach to extrapolate toxicological data from the chemicals we tested in order to predict their relative toxicity based on shared physicochemical characteristics. Clustering is a machine learning technique that involves the grouping of data points based on shared properties (Kriegel et al., 2011; Färber et al., 2010). We analyzed our accrued toxicity data in order to develop a protocol to potentially predict the general toxicity of untested chemicals based on their physicochemical properties. Chemicals were grouped based on their combined assay results. Physicochemical properties (solubility, density, molecular weight, listed Safety Data Sheet hazards, functional groups, and canonical SMILES) were then used to attempt to predict the group classification of the chemicals to uncover any driving properties behind the groups that were determined from assay results (i.e., properties that were potentially causing the toxicity effects seen in the assays). The simplified molecular-input line-entry system (SMILES) is a way of describing the structure of a molecule as a single line of text. (e.g., vanillin: COC1=C[C=CC[=C1]C=O]O).

Methods

*Clustering*

Average viability, lactate dehydrogenase (LDH), intracellular reactive oxygen species (ROS), and cytokine concentration (IL-1β, IL-6, IL-8, and TNF-$\alpha$) were calculated for each chemical and stratified by cell type and time point. Groupings of the 30 chemicals plus the propylene glycol, vegetable glycerin (PG/VG) vehicle control (phosphate buffer solution [PBS] control and positive control excluded) were determined based on treatment averages from all seven assays using Hierarchical Clustering (Euclidean distance as metric). Human bronchial epithelial cells (BEAS-2B) cell type dendrograms were cut to form four groups of chemicals with similar assay patterns, and THP-1 (activated and naïve) dendrograms were cut to form three groups of chemicals. The number of groups for each cell type was determined by cutting the dendrogram at the largest height that still resulted in distinct groupings of chemicals based on toxicity. Grouping in this manner allowed the separation of different "classes" of toxicity (e.g., high potency versus medium or low potency) within the chemicals without creating overly specific groupings for investigation of any commonalities amongst the physicochemical properties. All clustering analyses were performed at the highest dose group for each cell type-chemical treatment combination.

*Modeling*

Replicates (50, 100, and 150) for each chemical–assay–time point–cell type combination were randomly generated from a Normal distribution with mean and standard deviation equal to the corresponding sample mean and sample standard deviation. For example, the sample mean and sample standard deviation results from each assay from vehicle control treatment of BEAS-2B at the 4-hour time point were used as the mean and standard deviation parameters of the

Normal distribution to randomly generate 50, 100, and 150 data points per assay. This was repeated for each cell type–time point–chemical treatment combination for all assays. Physicochemical properties including molecular weight (g/mol), density (g/mL), solubility in $H_2O$ (mM), Safety Data Sheet (SDS) hazard classifications, functional group list, and canonical SMILES were repeated uniformly across replicates within each chemical. Data generation was performed from the sample statistics due the moderately-high dimensionality of the dataset once stratified by cell type and timepoint, which could result in biased or overstated modeling results due to the small size of the training dataset once split for testing versus training. Three replication sizes (50, 100, and 150) were used to test the sensitivity of modeling results based on replication number.

Each set of replicated data was randomly split 80/20 for training/testing, respectively. The physicochemical property data were used in a classification Random Forest (number of trees = 500, seed set to 1234) to classify materials into groups corresponding to the groupings that were determined from the stratified Hierarchical Clustering of the assay averages. Variable importance was assessed by Mean Decrease in Accuracy and Mean Decrease in Gini Index. Results displayed are for sets from 150 replications since results were consistent across replication number.

All clustering and modeling analyses were conducted in R version 3.6.0 using randomForest package for modeling [34,35].

## Results

Across all cell types and time points, decanal, eugenol, and nonanal consistently were placed in the most potent groupings (Figures S9-S11). Additionally, Alpha-pinene [(-)-a-Pinene] and Limonene [(R)-(+)-Limonene] were commonly grouped in the high/moderate toxicity grouping (ref fig/table of clustering results). Random forest results showed that canonical SMILES and functional groups were the most important physicochemical properties in accurately (100% accuracy in all cell types and time points) predicting a compound's toxicity grouping, which was determined via clustering based on average assay results. Canonical SMILES and functional groups were also the top two variables of importance when Mean Decrease in Gini Index was evaluated. Other physicochemical properties, such as solubility, density, molecular weight, and SDS hazard were consistently less important and did not display a uniform trend for prediction of toxicity grouping across cell types and time points. Common functional groups amongst the chemicals within the most potent groupings include benzenes and aldehydes, as well as isoprenes and methyl groups.

After grouping flavoring chemicals by their toxicity and analyzing by solubility, molecular weight, density, functional groups, and canonical SMILES, our model suggests that the most effective method to accurately predict the toxicity of a flavoring chemical is by assessing the canonical SMILE signature for effects on BEAS-2B cells and functional group for THP-1 cells. The flavoring chemicals which appear to affect cells the most fall into the categories of aldehydes with large carbon chains attached, compounds containing benzene rings, and chemicals classified as monoterpenes, which contain two isoprene groups.

A.) BEAS-2B

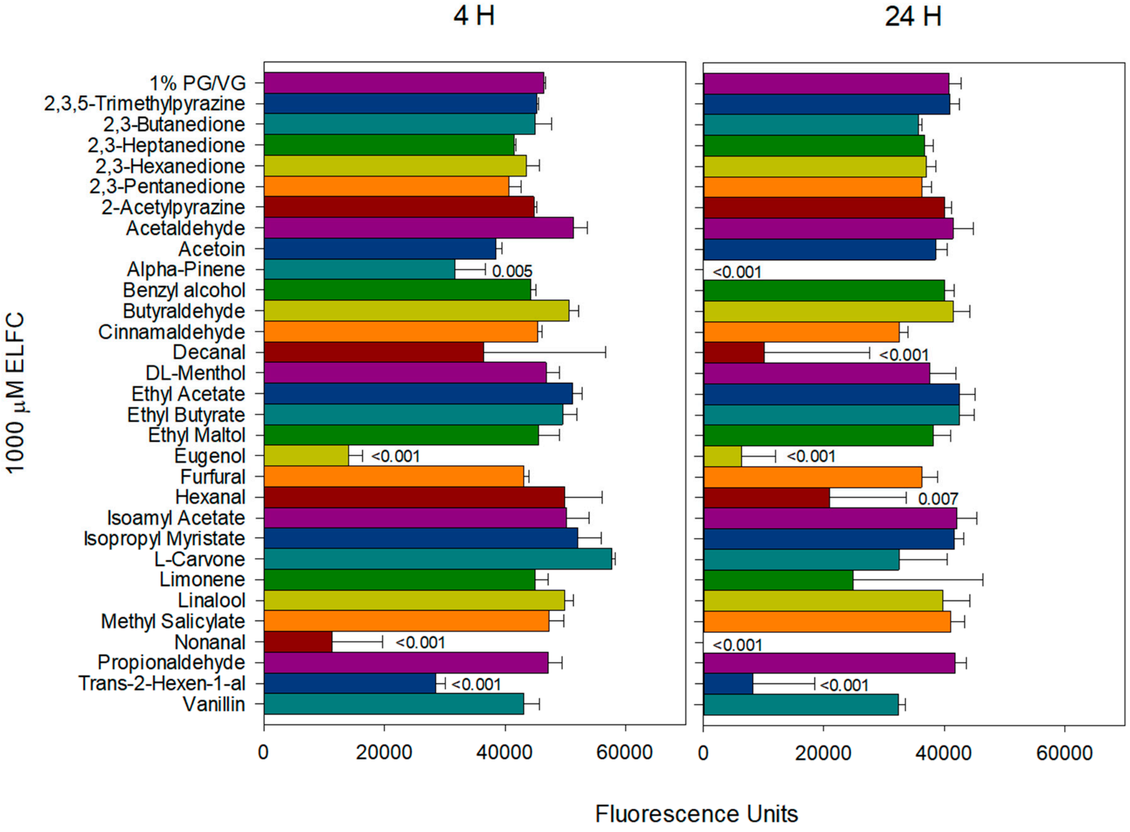
4 H
24 H
1000 µM ELFC
1% PG/VG
2,3,5-Trimethylpyrazine
2,3-Butanedione
2,3-Heptanedione
2,3-Hexanedione
2,3-Pentanedione
2-Acetylpyrazine
Acetaldehyde
Acetoin
Alpha-Pinene
Benzyl alcohol
Butyraldehyde
Cinnamaldehyde
Decanal
DL-Menthol
Ethyl Acetate
Ethyl Butyrate
Ethyl Maltol
Eugenol
Furfural
Hexanal
Isoamyl Acetate
Isopropyl Myristate
L-Carvone
Limonene
Linalool
Methyl Salicylate
Nonanal
Propionaldehyde
Trans-2-Hexen-1-al
Vanillin
0.005
<0.001
<0.001
<0.001
<0.001
<0.001
0.007
<0.001
<0.001
<0.001
<0.001
0
20000
40000
60000
0
20000
40000
60000
Fluorescence Units

B.) Naïve THP-1

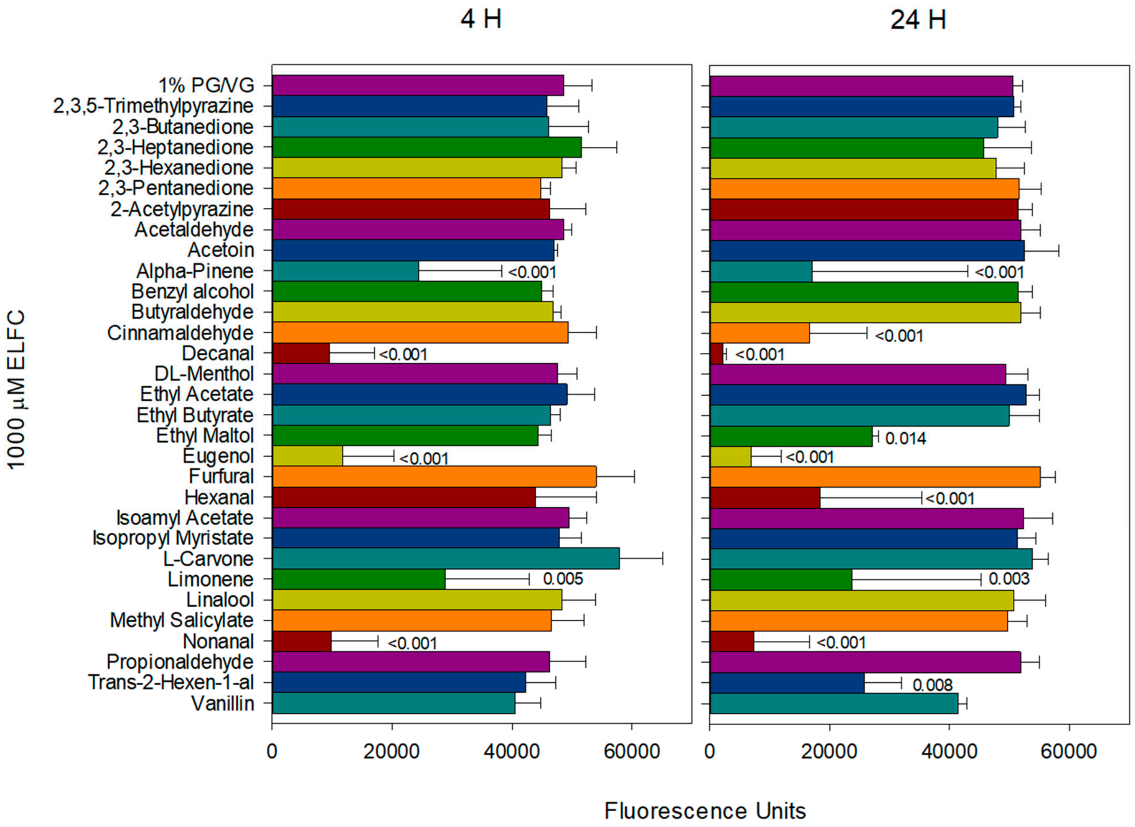

4 H
24 H
1000 µM ELFC
1% PG/VG
2,3,5-Trimethylpyrazine
2,3-Butanedione
2,3-Heptanedione
2,3-Hexanedione
2,3-Pentanedione
2-Acetylpyrazine
Acetaldehyde
Acetoin
Alpha-Pinene
Benzyl alcohol
Butyraldehyde
Cinnamaldehyde
Decanal
DL-Menthol
Ethyl Acetate
Ethyl Butyrate
Ethyl Maltol
Eugenol
Furfural
Hexanal
Isoamyl Acetate
Isopropyl Myristate
L-Carvone
Limonene
Linalool
Methyl Salicylate
Nonanal
Propionaldehyde
Trans-2-Hexen-1-al
Vanillin
<0.001
<0.001
<0.001
0.005
<0.001
<0.001
<0.001
<0.001
0.014
<0.001
<0.001
0.003
<0.001
0.008
0
20000
40000
60000
Fluorescence Units

C.) Activated THP-1

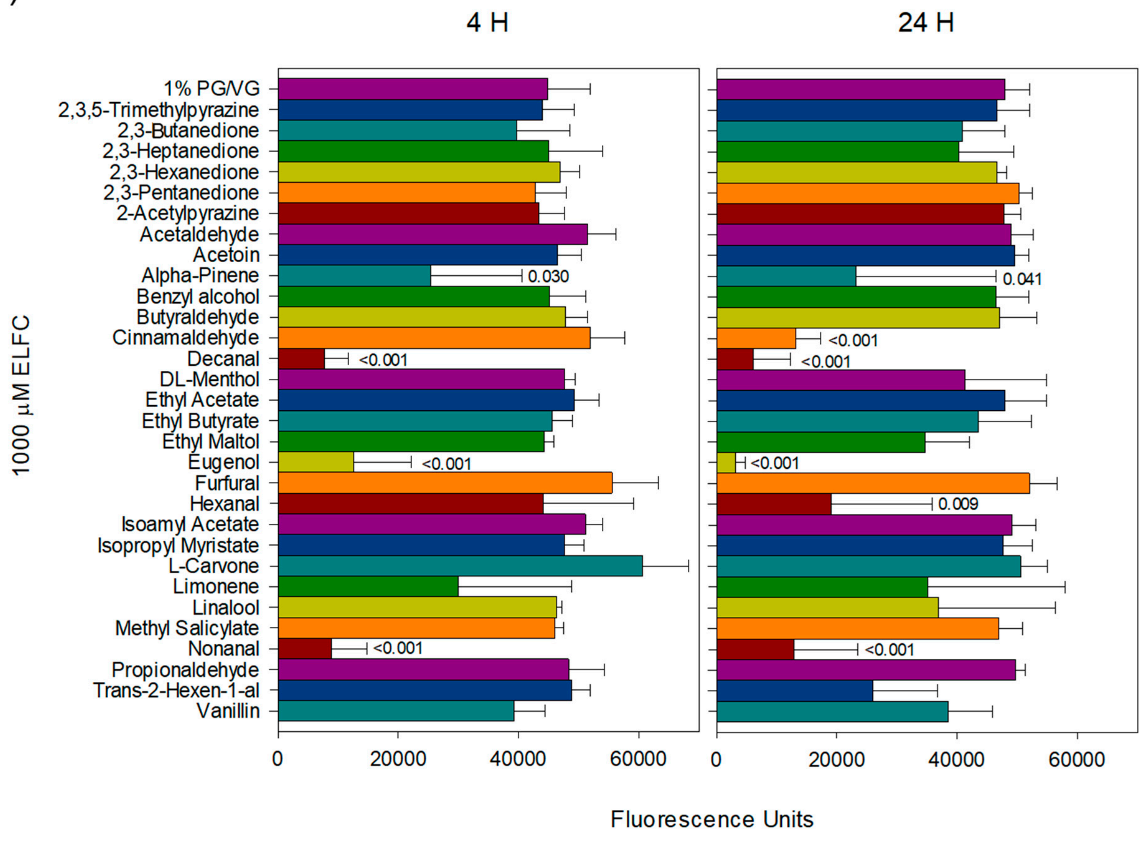


**Figure S1.** Cell Viability

A.) BEAS-2B

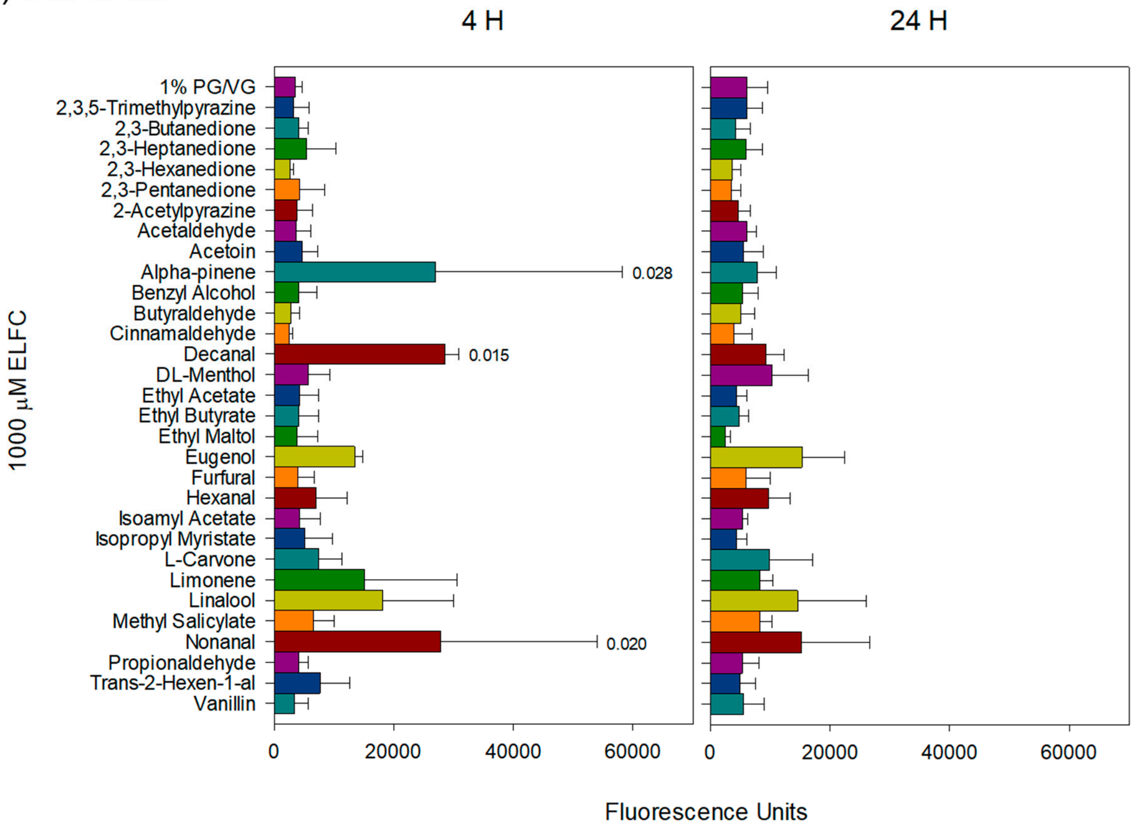

4 H
24 H
1000 μM ELFC
1% PG/VG
2,3,5-Trimethylpyrazine
2,3-Butanedione
2,3-Heptanedione
2,3-Hexanedione
2,3-Pentanedione
2-Acetylpyrazine
Acetaldehyde
Acetoin
Alpha-pinene
Benzyl Alcohol
Butyraldehyde
Cinnamaldehyde
Decanal
DL-Menthol
Ethyl Acetate
Ethyl Butyrate
Ethyl Maltol
Eugenol
Furfural
Hexanal
Isoamyl Acetate
Isopropyl Myristate
L-Carvone
Limonene
Linalool
Methyl Salicylate
Nonanal
Propionaldehyde
Trans-2-Hexen-1-al
Vanillin
0.028
0.015
0.020
0
20000
40000
60000
Fluorescence Units

B.) Naïve THP-1

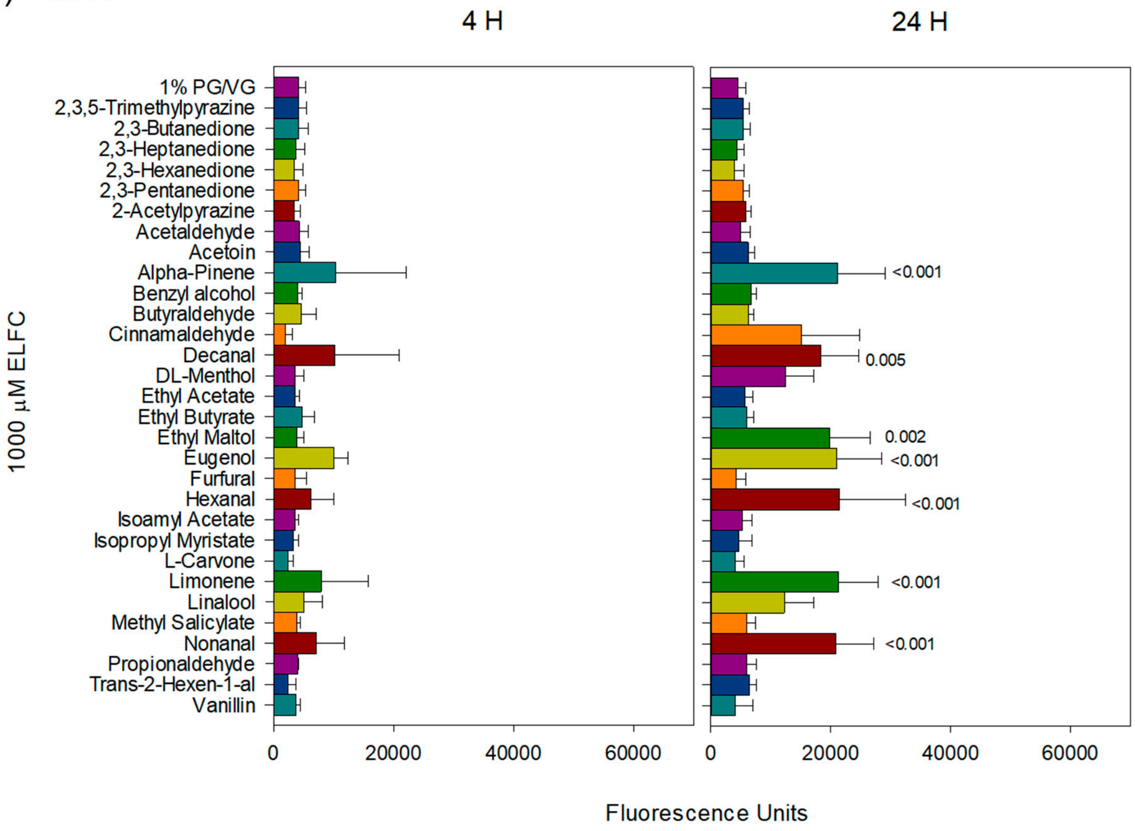
4 H
24 H
1000 μM ELFC
1% PG/VG
2,3,5-Trimethylpyrazine
2,3-Butanedione
2,3-Heptanedione
2,3-Hexanedione
2,3-Pentanedione
2-Acetylpyrazine
Acetaldehyde
Acetoin
Alpha-Pinene
Benzyl alcohol
Butyraldehyde
Cinnamaldehyde
Decanal
DL-Menthol
Ethyl Acetate
Ethyl Butyrate
Ethyl Maltol
Eugenol
Furfural
Hexanal
Isoamyl Acetate
Isopropyl Myristate
L-Carvone
Limonene
Linalool
Methyl Salicylate
Nonanal
Propionaldehyde
Trans-2-Hexen-1-al
Vanillin
<0.001
0.005
0.002
<0.001
<0.001
<0.001
<0.001
<0.001
0
20000
40000
60000
Fluorescence Units

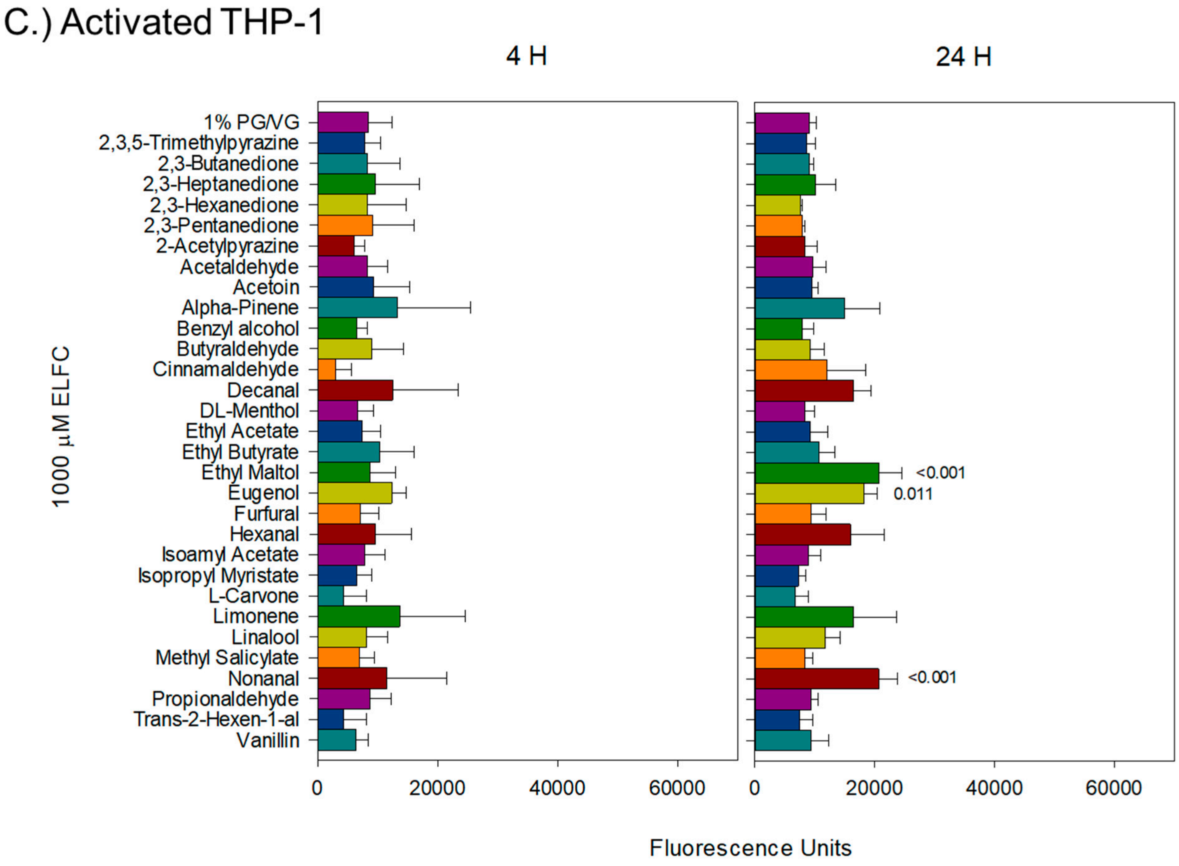


**Figure S2.** LDH

A.) BEAS-2B

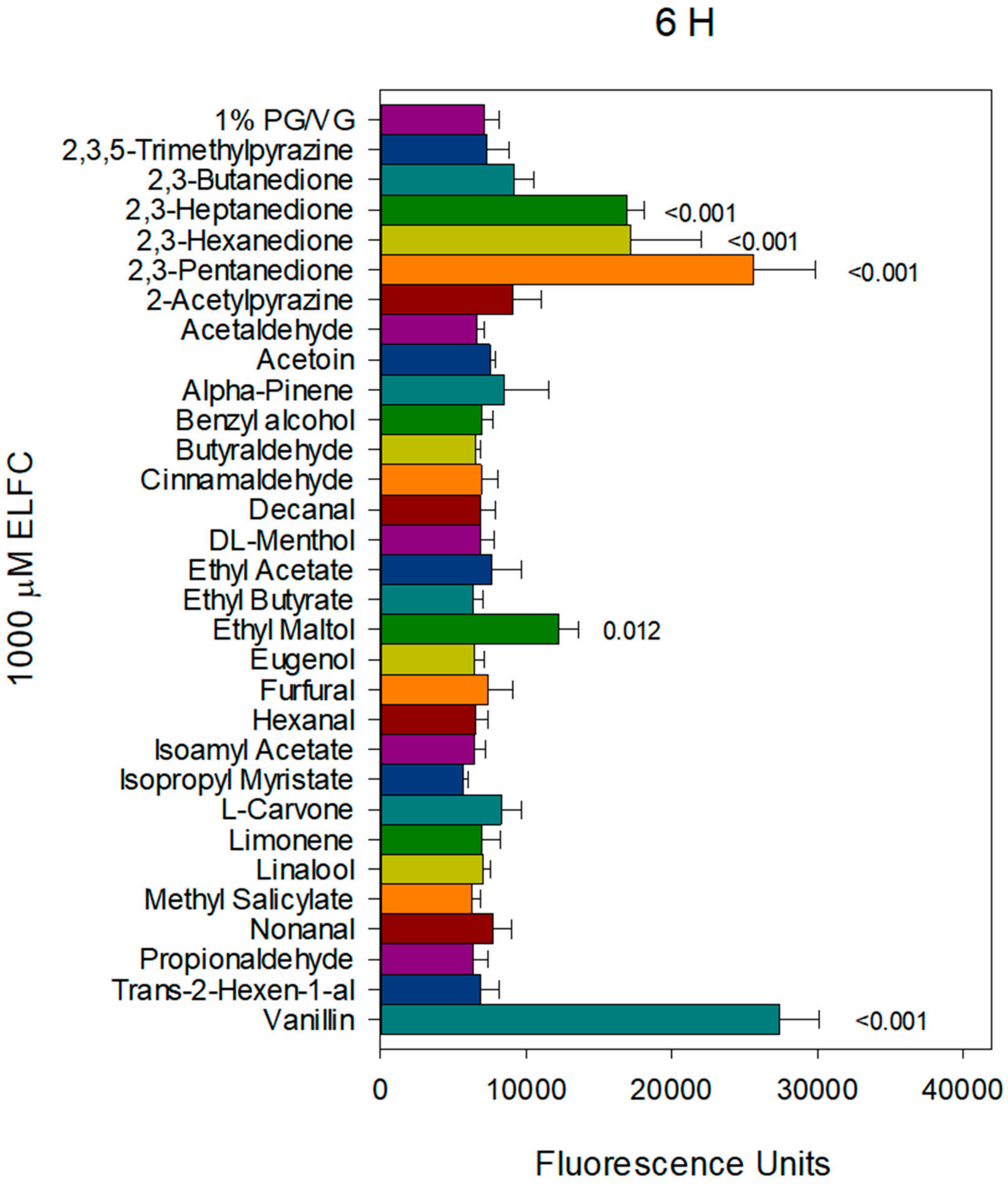
6 H
1% PG/VG
2,3,5-Trimethylpyrazine
2,3-Butanedione
2,3-Heptanedione
2,3-Hexanedione
2,3-Pentanedione
2-Acetylpyrazine
Acetaldehyde
Acetoin
Alpha-Pinene
Benzyl alcohol
Butyraldehyde
Cinnamaldehyde
Decanal
DL-Menthol
Ethyl Acetate
Ethyl Butyrate
Ethyl Maltol
Eugenol
Furfural
Hexanal
Isoamyl Acetate
Isopropyl Myristate
L-Carvone
Limonene
Linalool
Methyl Salicylate
Nonanal
Propionaldehyde
Trans-2-Hexen-1-al
Vanillin
<0.001
<0.001
<0.001
0.012
<0.001
1000 μM ELFC
0
10000
20000
30000
40000
Fluorescence Units

B.) Naïve THP-1

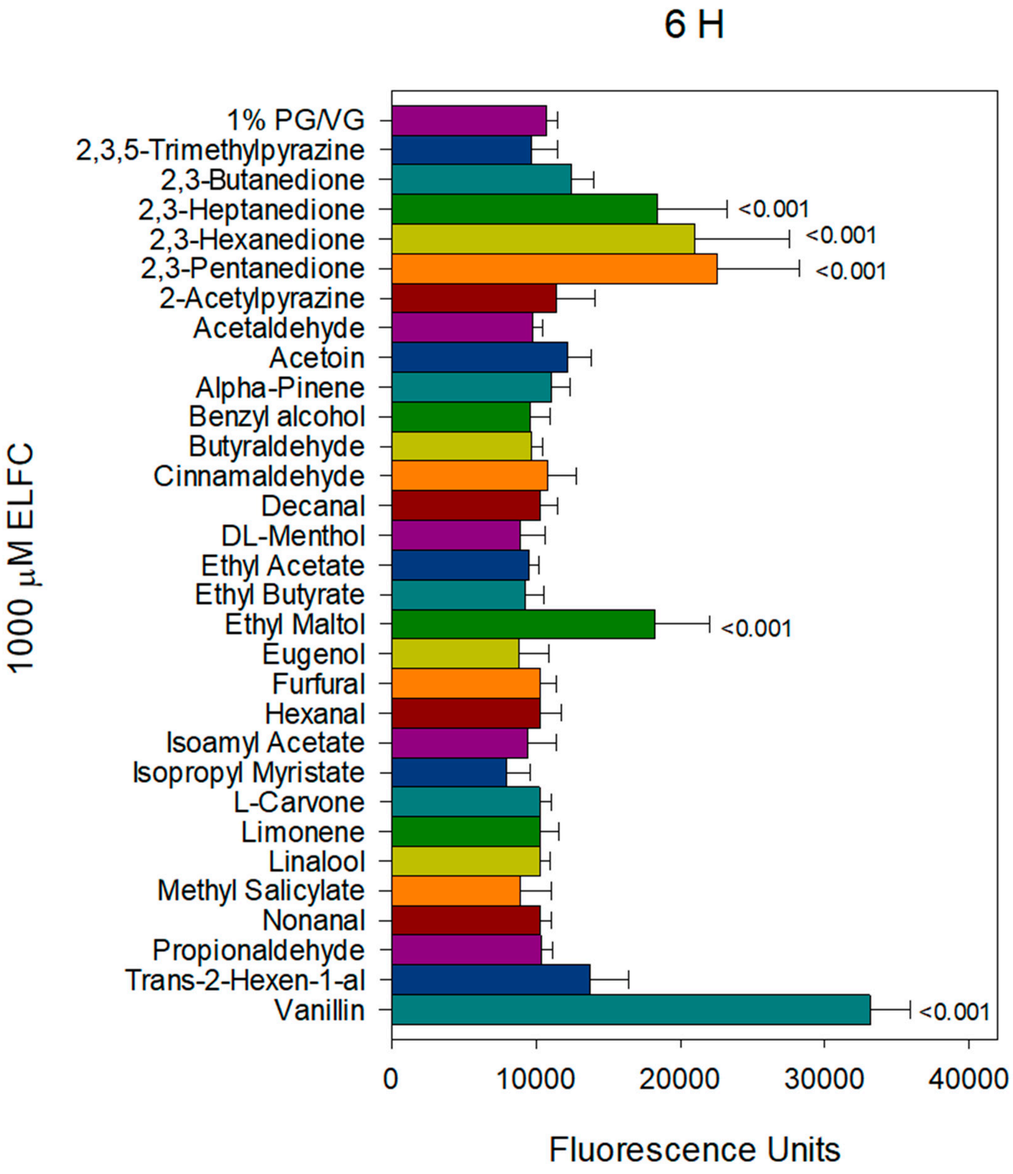

6 H
1000 μM ELFC
1% PG/VG
2,3,5-Trimethylpyrazine
2,3-Butanedione
2,3-Heptanedione
2,3-Hexanedione
2,3-Pentanedione
2-Acetylpyrazine
Acetaldehyde
Acetoin
Alpha-Pinene
Benzyl alcohol
Butyraldehyde
Cinnamaldehyde
Decanal
DL-Menthol
Ethyl Acetate
Ethyl Butyrate
Ethyl Maltol
Eugenol
Furfural
Hexanal
Isoamyl Acetate
Isopropyl Myristate
L-Carvone
Limonene
Linalool
Methyl Salicylate
Nonanal
Propionaldehyde
Trans-2-Hexen-1-al
Vanillin
<0.001
<0.001
<0.001
<0.001
<0.001
0
10000
20000
30000
40000
Fluorescence Units

C.) Activated THP-1

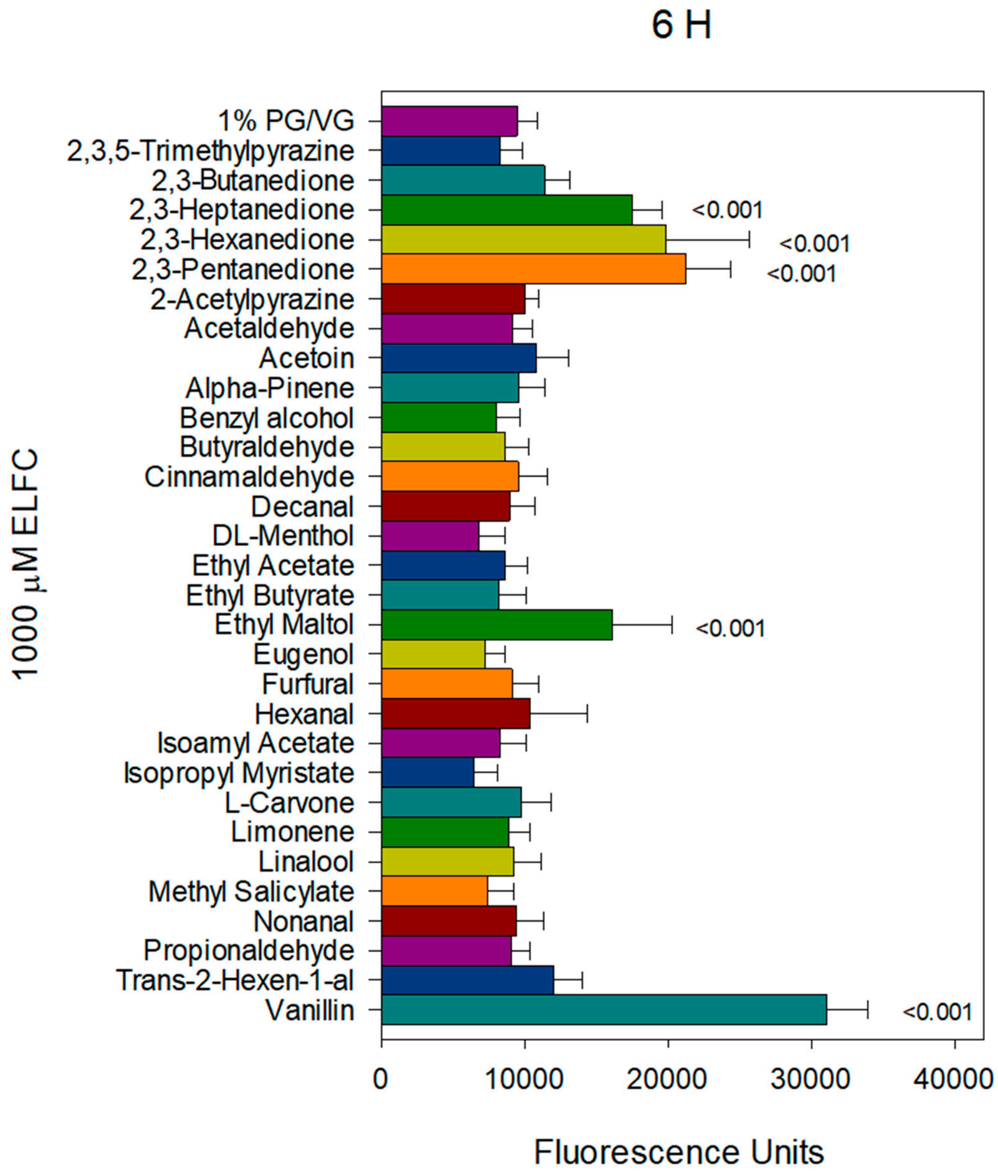


**Figure S3.** Intracellular ROS

A.) BEAS-2B

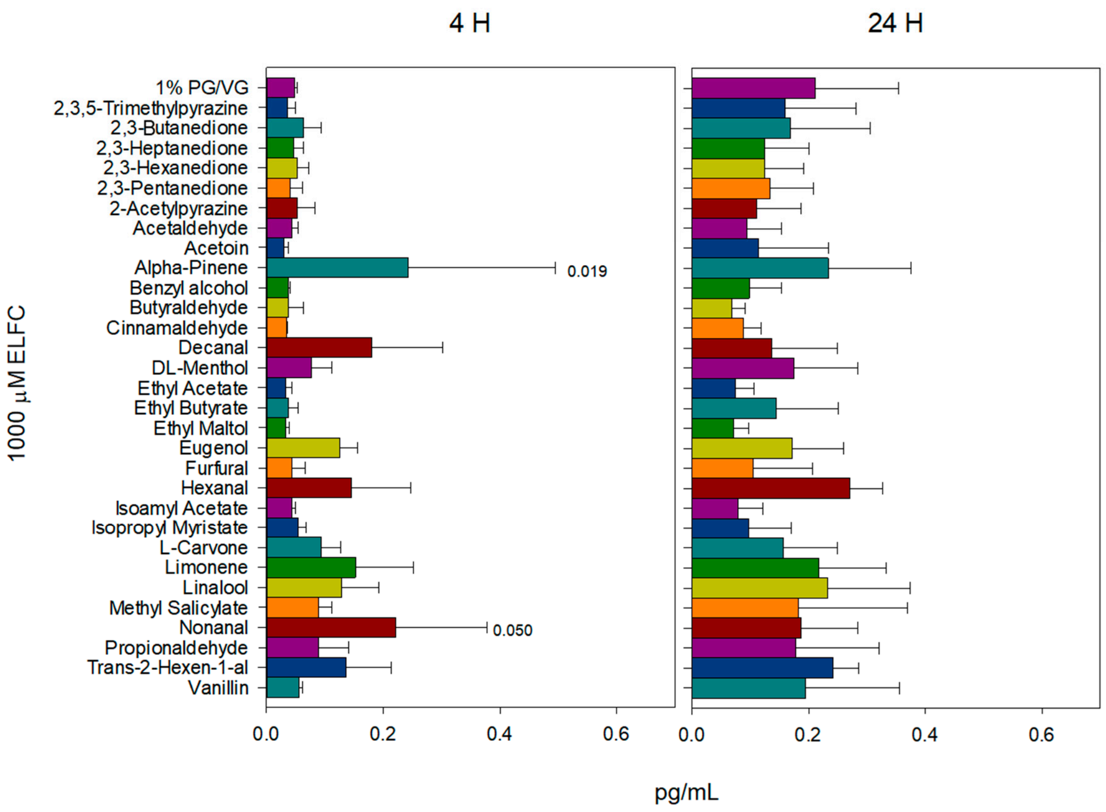

4 H
24 H
1% PG/VG
2,3,5-Trimethylpyrazine
2,3-Butanedione
2,3-Heptanedione
2,3-Hexanedione
2,3-Pentanedione
2-Acetylpyrazine
Acetaldehyde
Acetoin
Alpha-Pinene
Benzyl alcohol
Butyraldehyde
Cinnamaldehyde
Decanal
DL-Menthol
Ethyl Acetate
Ethyl Butyrate
Ethyl Maltol
Eugenol
Furfural
Hexanal
Isoamyl Acetate
Isopropyl Myristate
L-Carvone
Limonene
Linalool
Methyl Salicylate
Nonanal
Propionaldehyde
Trans-2-Hexen-1-al
Vanillin
0.019
0.050
1000 μM ELFC
0.0
0.2
0.4
0.6
pg/mL

B.) Naïve THP-1

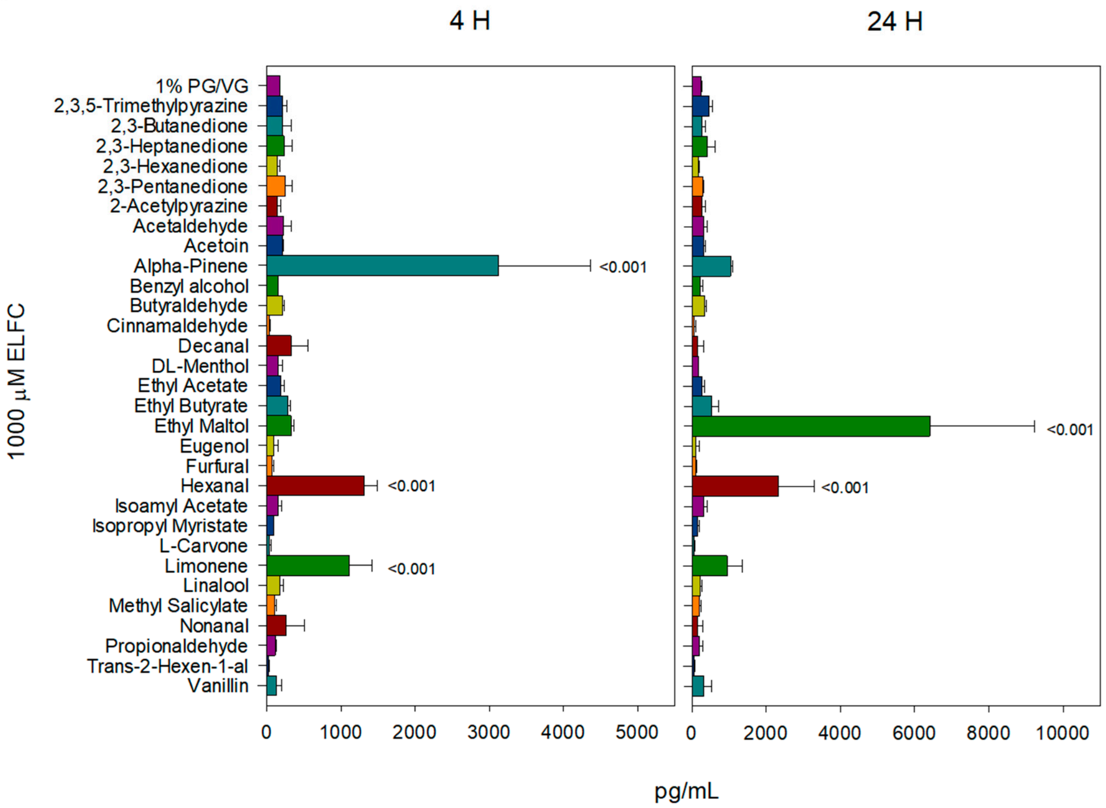
4 H
24 H
1000 μM ELFC
1% PG/VG
2,3,5-Trimethylpyrazine
2,3-Butanedione
2,3-Heptanedione
2,3-Hexanedione
2,3-Pentanedione
2-Acetylpyrazine
Acetaldehyde
Acetoin
Alpha-Pinene
Benzyl alcohol
Butyraldehyde
Cinnamaldehyde
Decanal
DL-Menthol
Ethyl Acetate
Ethyl Butyrate
Ethyl Maltol
Eugenol
Furfural
Hexanal
Isoamyl Acetate
Isopropyl Myristate
L-Carvone
Limonene
Linalool
Methyl Salicylate
Nonanal
Propionaldehyde
Trans-2-Hexen-1-al
Vanillin
<0.001
<0.001
<0.001
<0.001
<0.001
0
1000
2000
3000
4000
5000
0
2000
4000
6000
8000
10000
pg/mL

C.) Activated THP-1

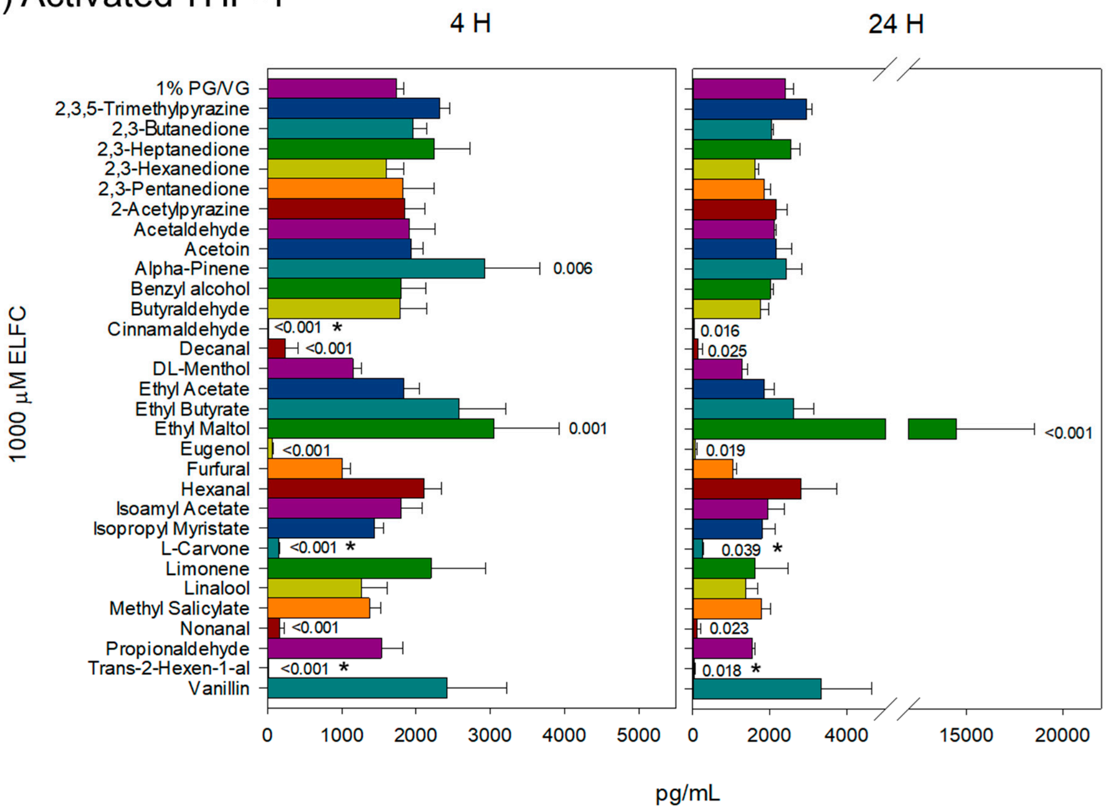


**Figure S4.** IL-1β

A.) BEAS-2B

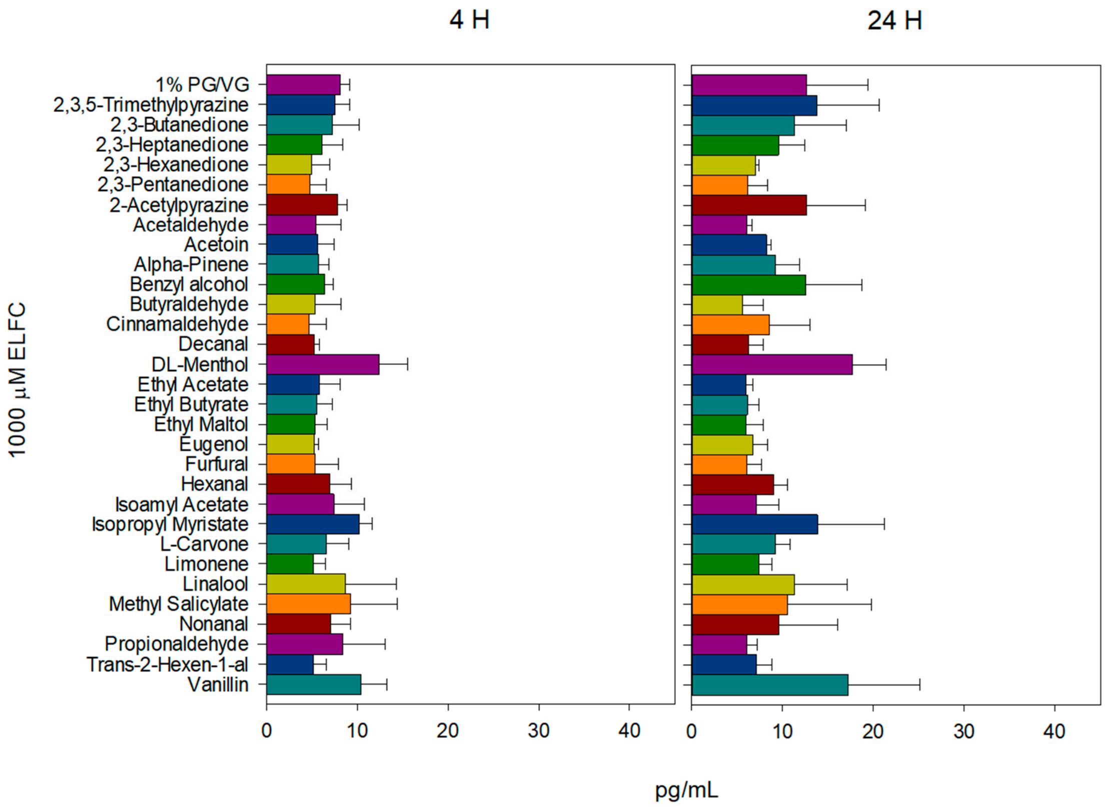
4 H
24 H
1000 μM ELFC
1% PG/VG
2,3,5-Trimethylpyrazine
2,3-Butanedione
2,3-Heptanedione
2,3-Hexanedione
2,3-Pentanedione
2-Acetylpyrazine
Acetaldehyde
Acetoin
Alpha-Pinene
Benzyl alcohol
Butyraldehyde
Cinnamaldehyde
Decanal
DL-Menthol
Ethyl Acetate
Ethyl Butyrate
Ethyl Maltol
Eugenol
Furfural
Hexanal
Isoamyl Acetate
Isopropyl Myristate
L-Carvone
Limonene
Linalool
Methyl Salicylate
Nonanal
Propionaldehyde
Trans-2-Hexen-1-al
Vanillin
0
10
20
30
40
pg/mL

B.) Naïve THP-1

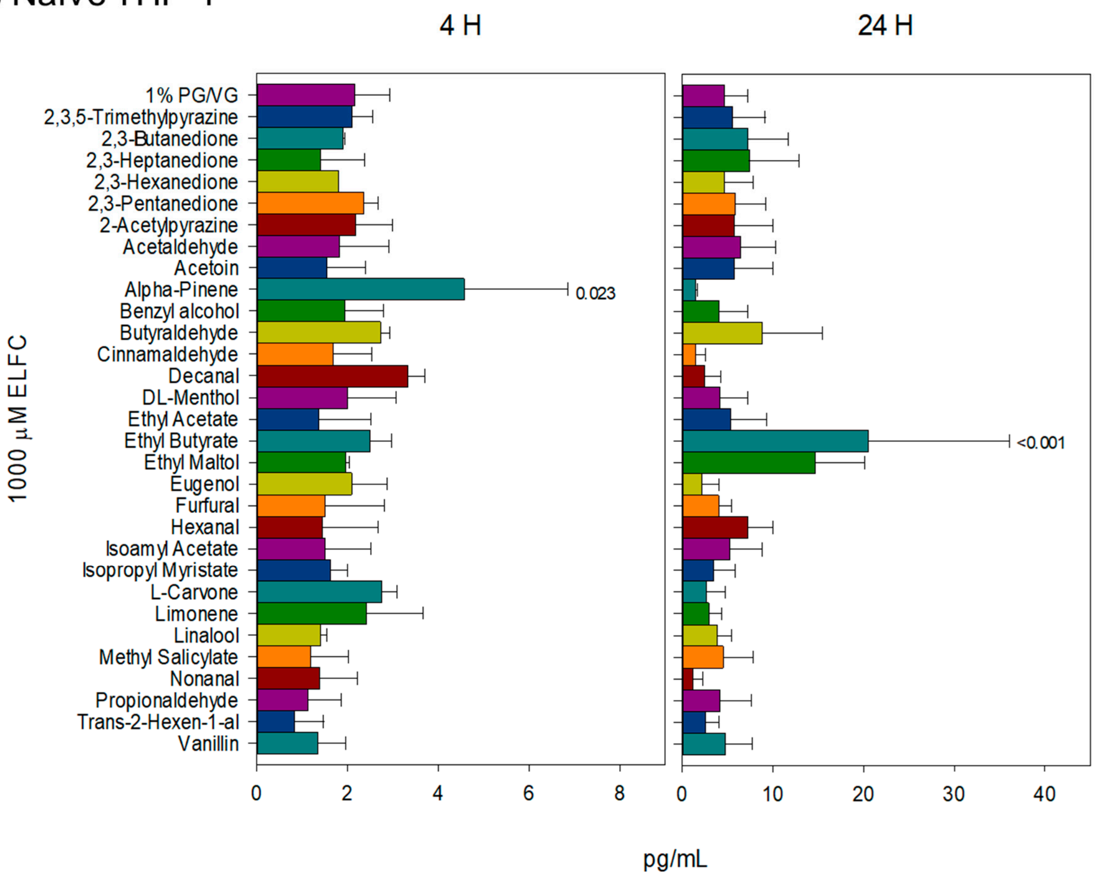
4 H
24 H
1% PG/VG
2,3,5-Trimethylpyrazine
2,3-Butanedione
2,3-Heptanedione
2,3-Hexanedione
2,3-Pentanedione
2-Acetylpyrazine
Acetaldehyde
Acetoin
Alpha-Pinene
Benzyl alcohol
Butyraldehyde
Cinnamaldehyde
Decanal
DL-Menthol
Ethyl Acetate
Ethyl Butyrate
Ethyl Maltol
Eugenol
Furfural
Hexanal
Isoamyl Acetate
Isopropyl Myristate
L-Carvone
Limonene
Linalool
Methyl Salicylate
Nonanal
Propionaldehyde
Trans-2-Hexen-1-al
Vanillin
0.023
<0.001
1000 μM ELFC
0 2 4 6 8
0 10 20 30 40
pg/mL

C.) Activated THP-1

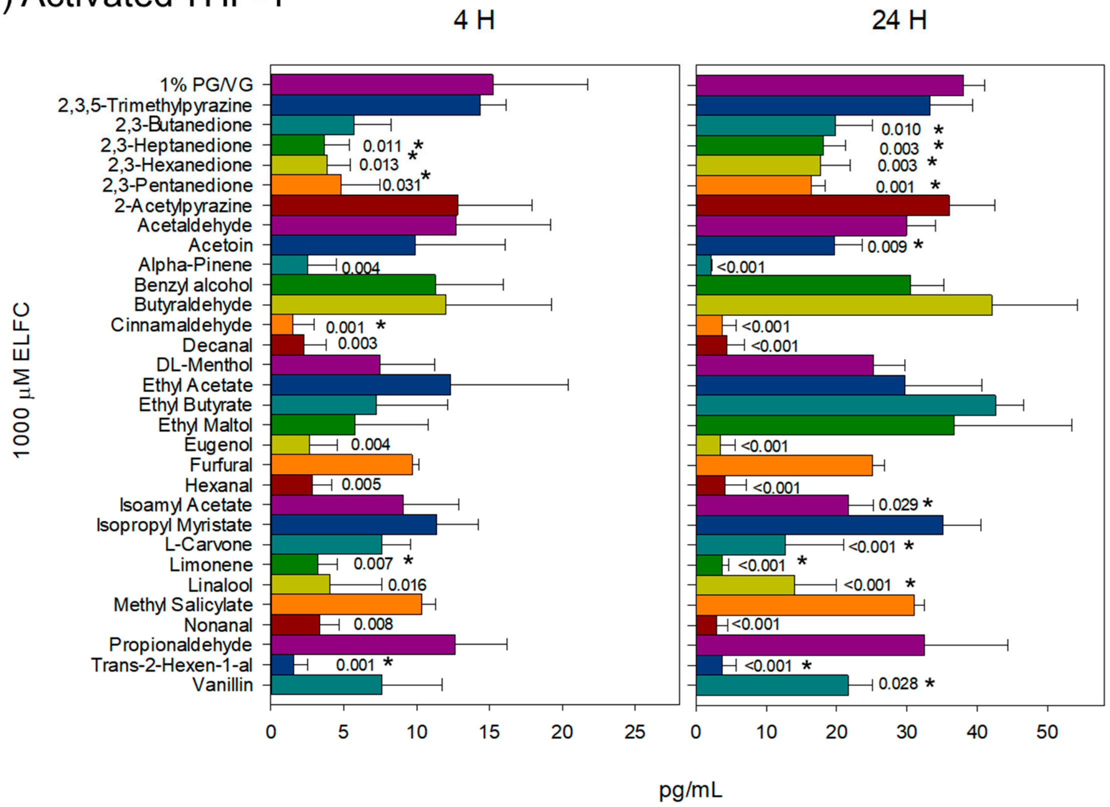


**Figure S5.** IL-6

A.) BEAS-2B

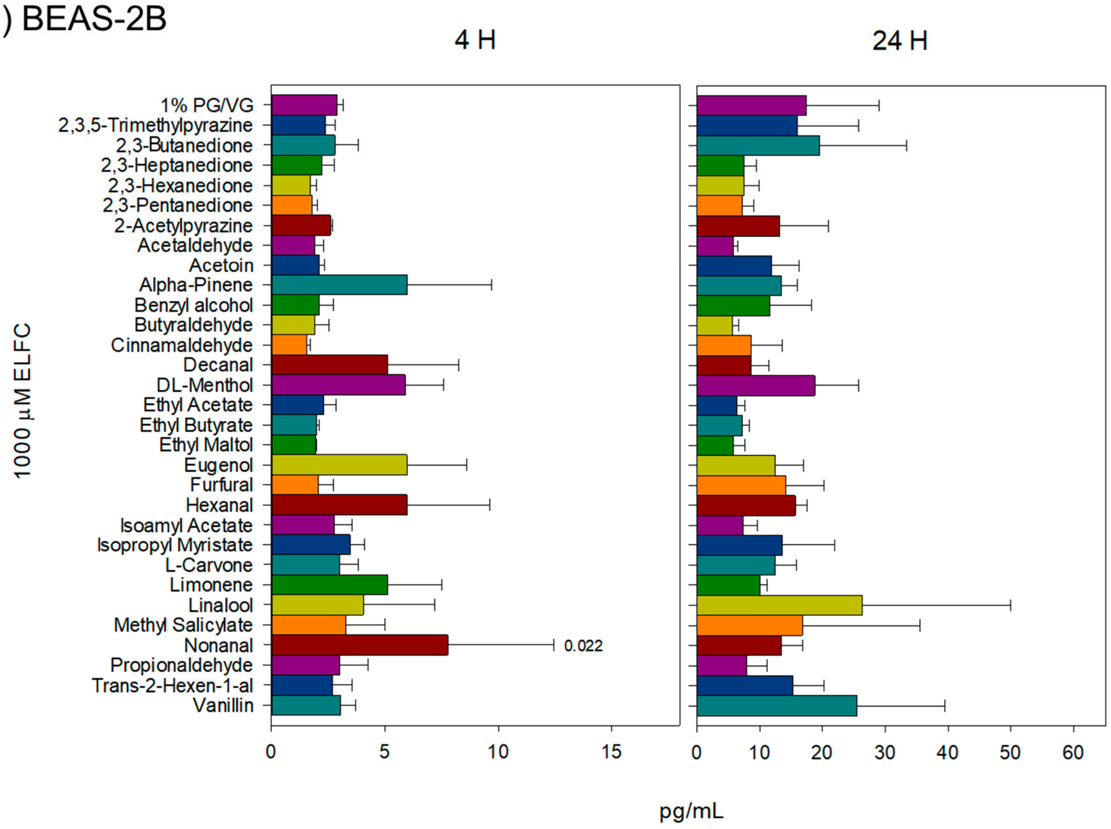

4 H
24 H
1000 µM ELFC
1% PG/VG
2,3,5-Trimethylpyrazine
2,3-Butanedione
2,3-Heptanedione
2,3-Hexanedione
2,3-Pentanedione
2-Acetylpyrazine
Acetaldehyde
Acetoin
Alpha-Pinene
Benzyl alcohol
Butyraldehyde
Cinnamaldehyde
Decanal
DL-Menthol
Ethyl Acetate
Ethyl Butyrate
Ethyl Maltol
Eugenol
Furfural
Hexanal
Isoamyl Acetate
Isopropyl Myristate
L-Carvone
Limonene
Linalool
Methyl Salicylate
Nonanal
Propionaldehyde
Trans-2-Hexen-1-al
Vanillin
0.022
0
5
10
15
0
10
20
30
40
50
60
pg/mL

B.) Naïve THP-1

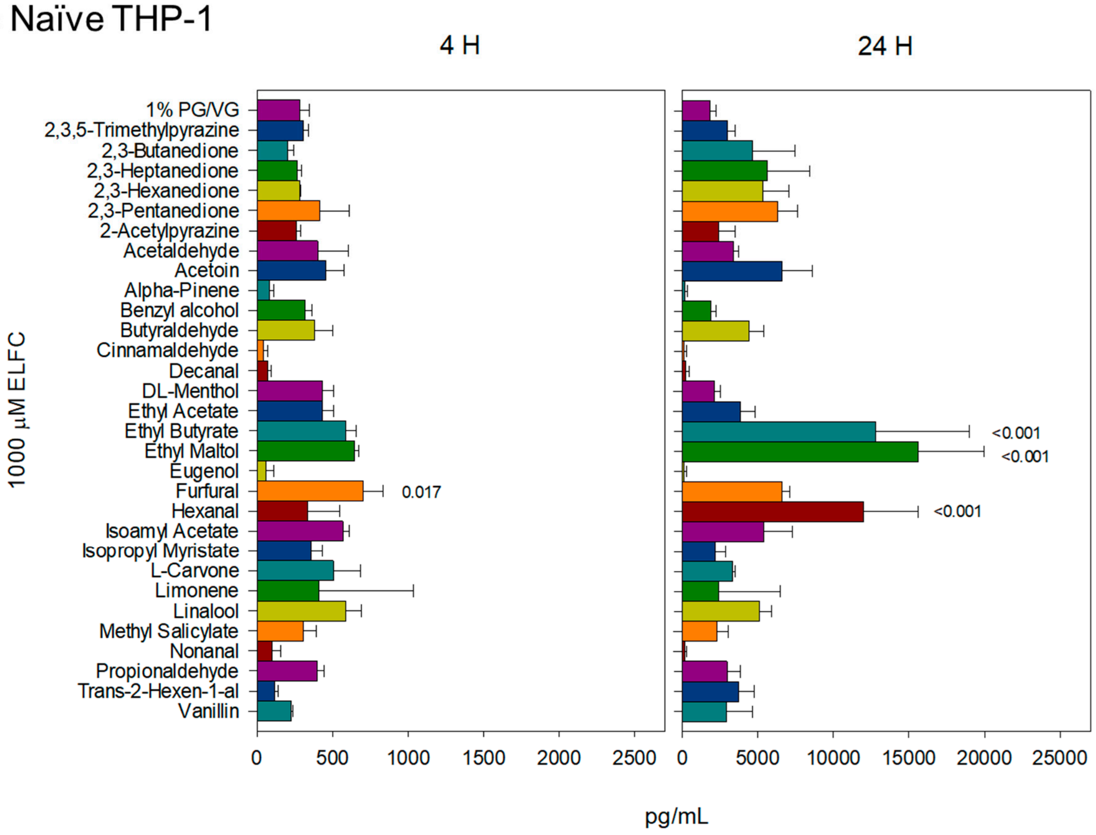

4 H
24 H
1000 μM ELFC
1% PG/VG
2,3,5-Trimethylpyrazine
2,3-Butanedione
2,3-Heptanedione
2,3-Hexanedione
2,3-Pentanedione
2-Acetylpyrazine
Acetaldehyde
Acetoin
Alpha-Pinene
Benzyl alcohol
Butyraldehyde
Cinnamaldehyde
Decanal
DL-Menthol
Ethyl Acetate
Ethyl Butyrate
Ethyl Maltol
Eugenol
Furfural
Hexanal
Isoamyl Acetate
Isopropyl Myristate
L-Carvone
Limonene
Linalool
Methyl Salicylate
Nonanal
Propionaldehyde
Trans-2-Hexen-1-al
Vanillin
0.017
<0.001
<0.001
<0.001
0
500
1000
1500
2000
2500
0
5000
10000
15000
20000
25000
pg/mL

C.) Activated THP-1

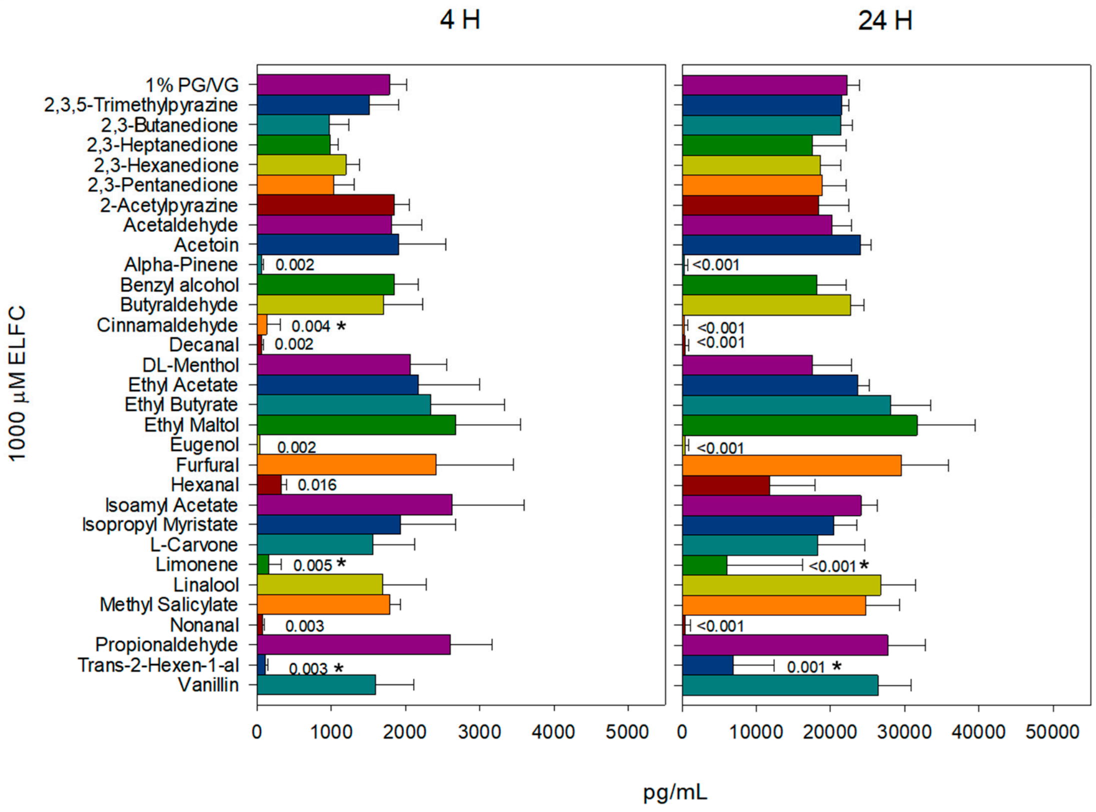


**Figure S6.** IL-8

A.) BEAS-2B

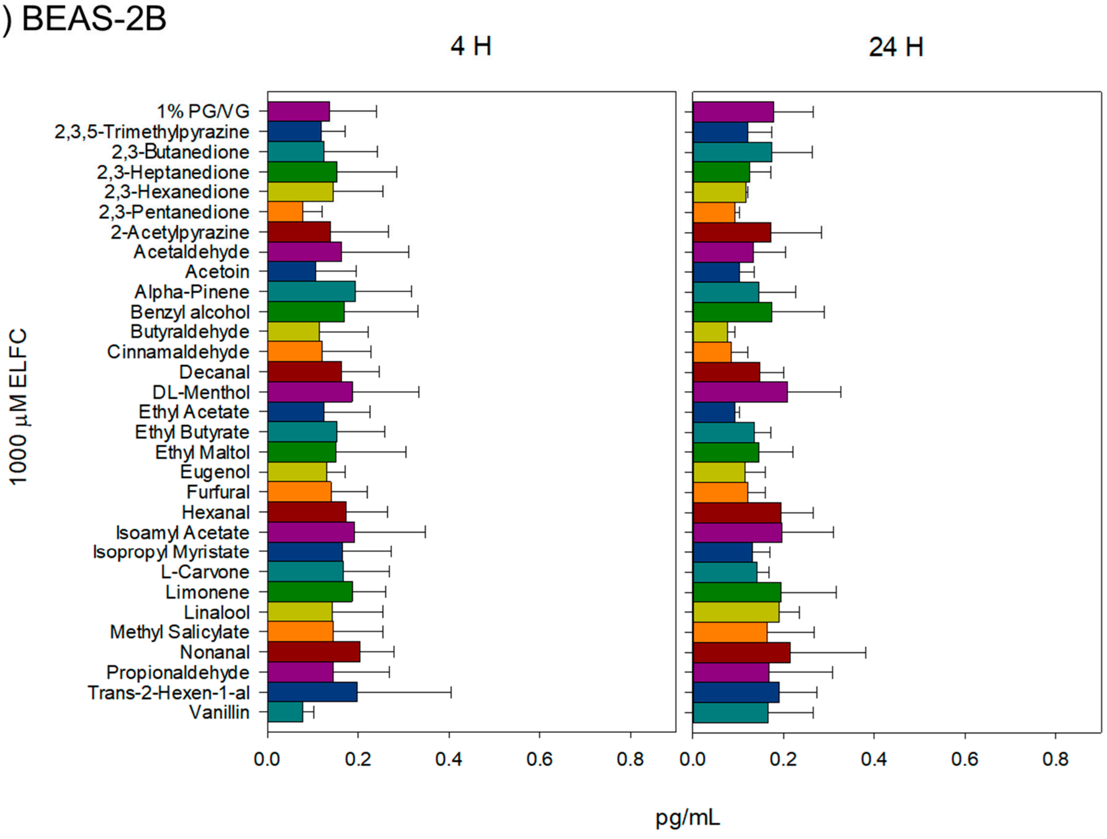
4 H
24 H
1000 µM ELFC
1% PG/VG
2,3,5-Trimethylpyrazine
2,3-Butanedione
2,3-Heptanedione
2,3-Hexanedione
2,3-Pentanedione
2-Acetylpyrazine
Acetaldehyde
Acetoin
Alpha-Pinene
Benzyl alcohol
Butyraldehyde
Cinnamaldehyde
Decanal
DL-Menthol
Ethyl Acetate
Ethyl Butyrate
Ethyl Maltol
Eugenol
Furfural
Hexanal
Isoamyl Acetate
Isopropyl Myristate
L-Carvone
Limonene
Linalool
Methyl Salicylate
Nonanal
Propionaldehyde
Trans-2-Hexen-1-al
Vanillin
0.0
0.2
0.4
0.6
0.8
pg/mL

B.) Naïve THP-1

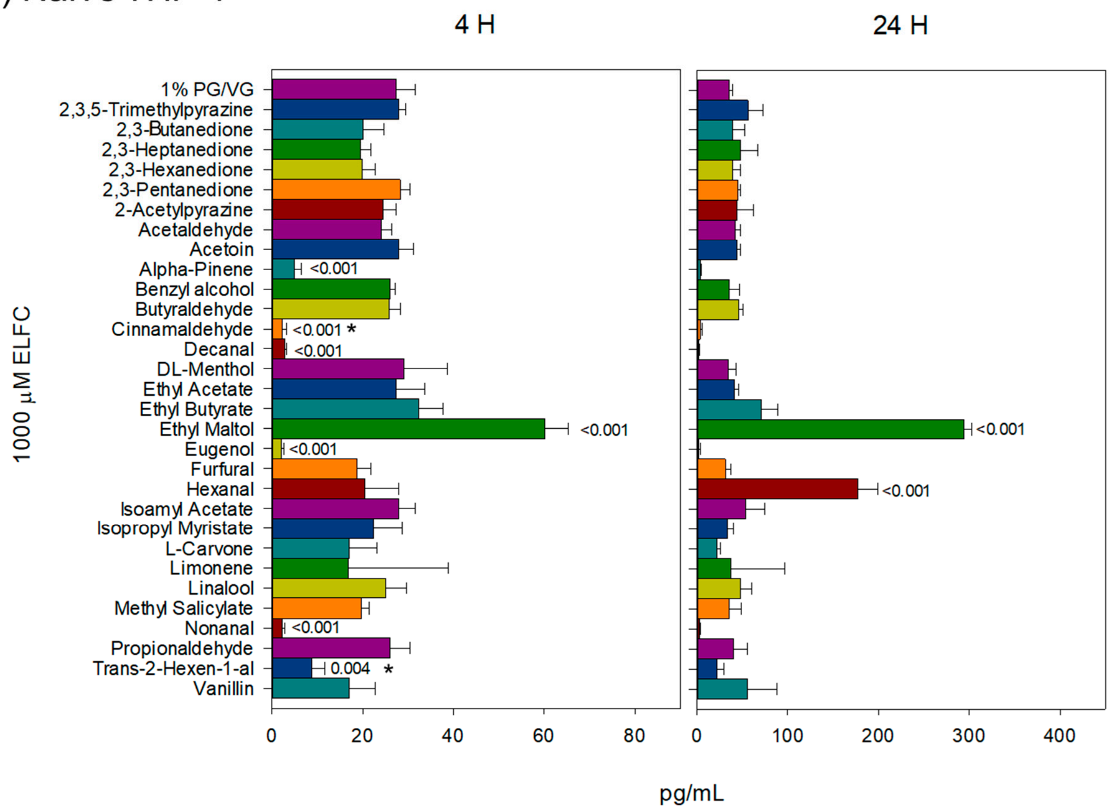

4 H
24 H
1000 μM ELFC
1% PG/VG
2,3,5-Trimethylpyrazine
2,3-Butanedione
2,3-Heptanedione
2,3-Hexanedione
2,3-Pentanedione
2-Acetylpyrazine
Acetaldehyde
Acetoin
Alpha-Pinene
Benzyl alcohol
Butyraldehyde
Cinnamaldehyde
Decanal
DL-Menthol
Ethyl Acetate
Ethyl Butyrate
Ethyl Maltol
Eugenol
Furfural
Hexanal
Isoamyl Acetate
Isopropyl Myristate
L-Carvone
Limonene
Linalool
Methyl Salicylate
Nonanal
Propionaldehyde
Trans-2-Hexen-1-al
Vanillin
<0.001
<0.001 *
<0.001
<0.001
<0.001
<0.001
0.004 *
<0.001
<0.001
0 20 40 60 80
0 100 200 300 400
pg/mL

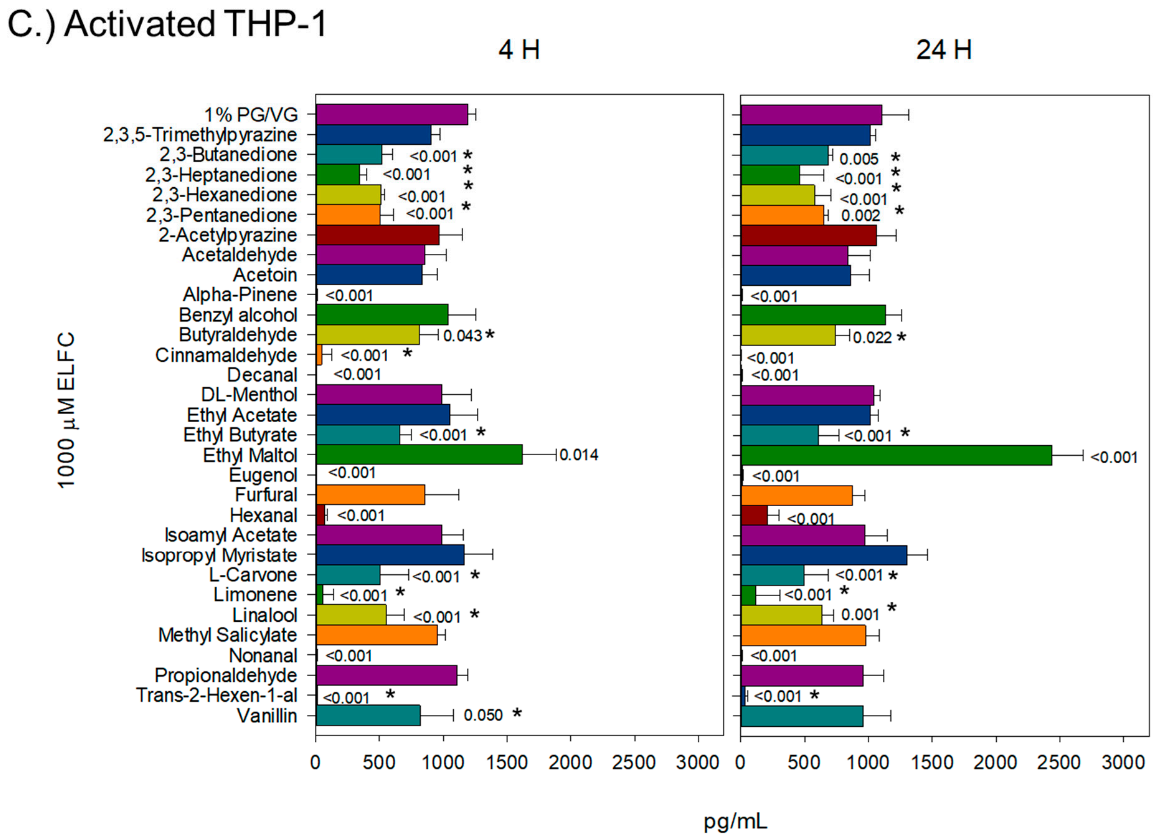


**Figure S7.** TNF-$\alpha$

A)

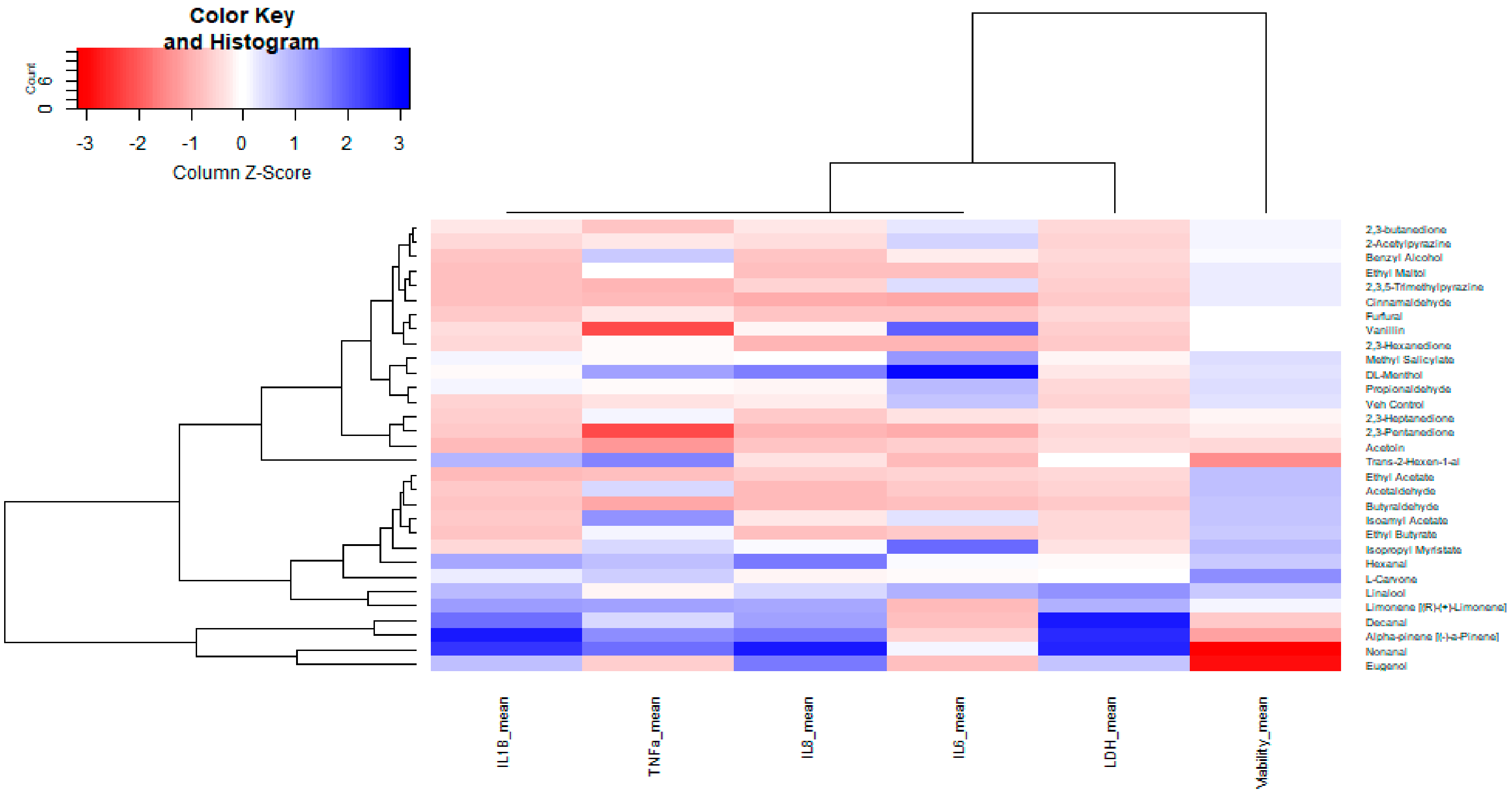

Color Key
and Histogram
Count
0
6
-3
-2
-1
0
1
2
3
Column Z-Score
2,3-butanedione
2-Acetylpyrazine
Benzyl Alcohol
Ethyl Maltol
2,3,5-Trimethylpyrazine
Cinnamaldehyde
Furfural
Vanillin
2,3-Hexanedione
Methyl Salicylate
DL-Menthol
Propionaldehyde
Veh Control
2,3-Heptanedione
2,3-Pentanedione
Acetoin
Trans-2-Hexen-1-al
Ethyl Acetate
Acetaldehyde
Butyraldehyde
Isoamyl Acetate
Ethyl Butyrate
Isopropyl Myristate
Hexanal
L-Carvone
Linalool
Limonene [(R)-(+)-Limonene]
Decanal
Alpha-pinene [(-)-a-Pinene]
Nonanal
Eugenol
IL1B_mean
TNFa_mean
IL8_mean
IL6_mean
LDH_mean
Viability_mean

B)

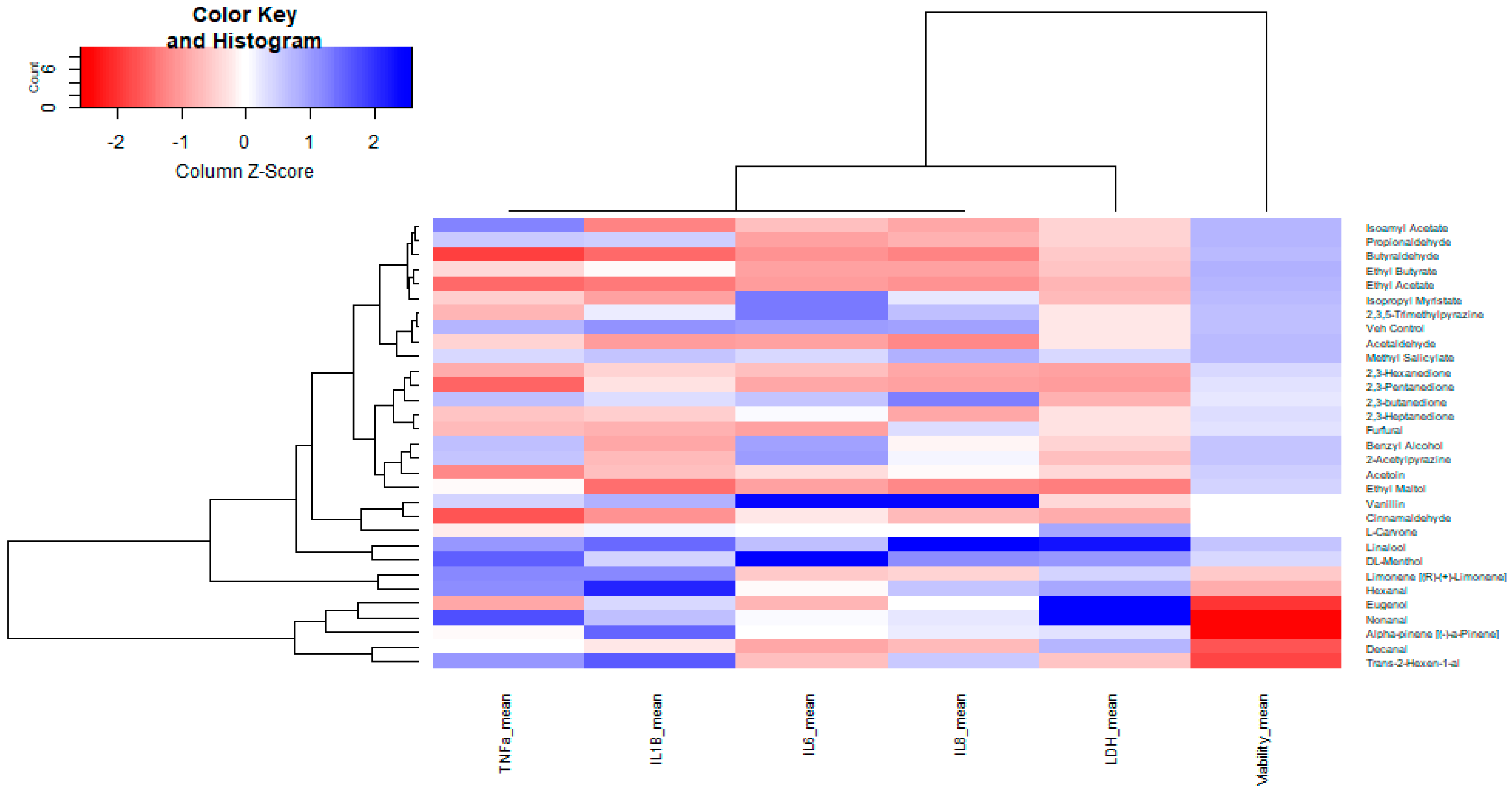


**Figure S8.** Heat map of clustering of toxicity tests in BEAS-2B. A) 4 hour B) 24 hour.

A)

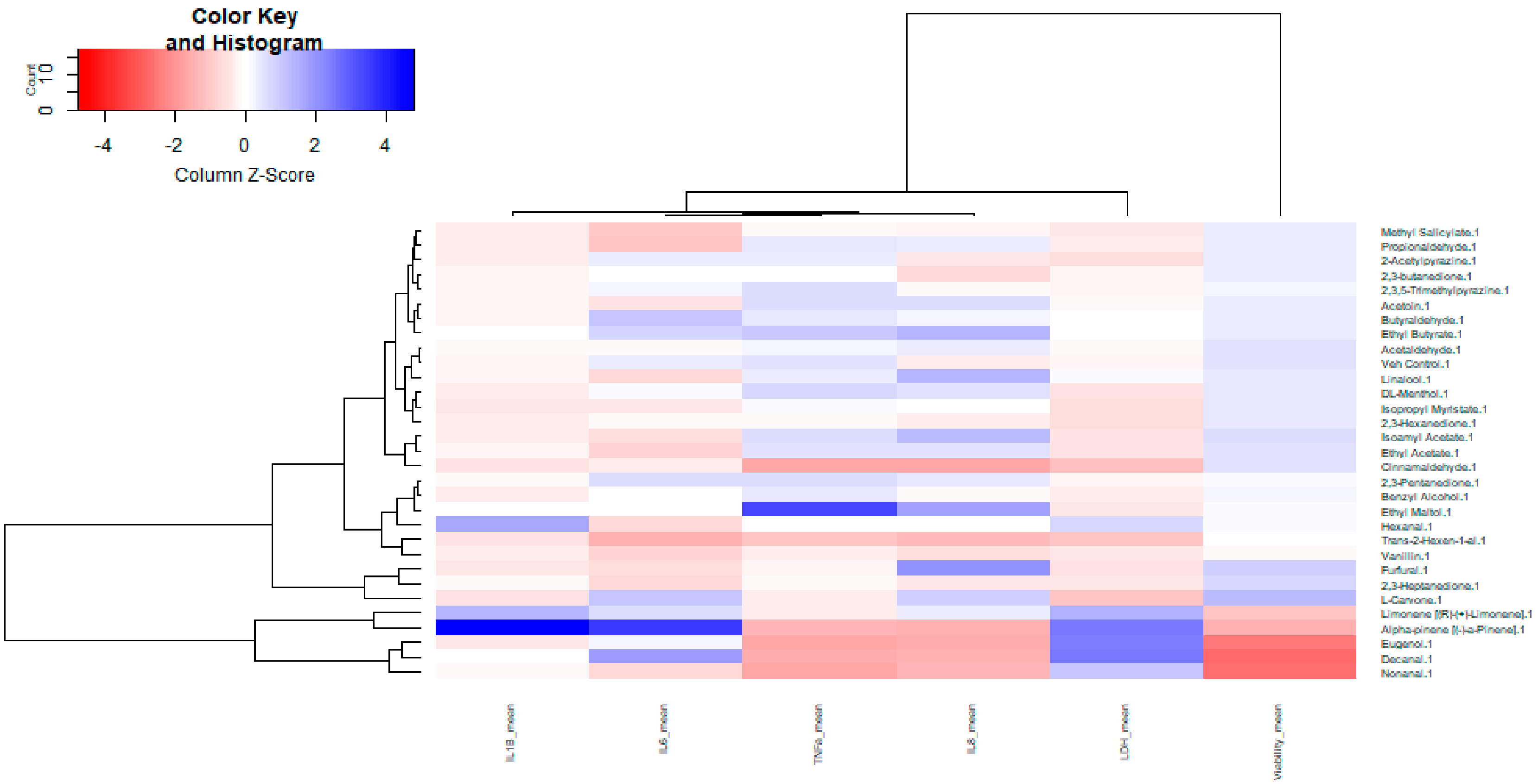
Color Key
and Histogram
Count
0
10
-4
-2
0
2
4
Column Z-Score
Methyl Salicylate.1
Propionaldehyde.1
2-Acetylpyrazine.1
2,3-butanedione.1
2,3,5-Trimethylpyrazine.1
Acetoin.1
Butyraldehyde.1
Ethyl Butyrate.1
Acetaldehyde.1
Veh Control.1
Linalool.1
DL-Menthol.1
Isopropyl Myristate.1
2,3-Hexanedione.1
Isoamyl Acetate.1
Ethyl Acetate.1
Cinnamaldehyde.1
2,3-Pentanedione.1
Benzyl Alcohol.1
Ethyl Maltol.1
Hexanal.1
Trans-2-Hexen-1-al.1
Vanillin.1
Furfural.1
2,3-Heptanedione.1
L-Carvone.1
Limonene [(R)-(+)-Limonene].1
Alpha-pinene [(-)-a-Pinene].1
Eugenol.1
Decanal.1
Nonanal.1
IL1B_mean
IL6_mean
TNFa_mean
IL8_mean
LDH_mean
Viability_mean

B)

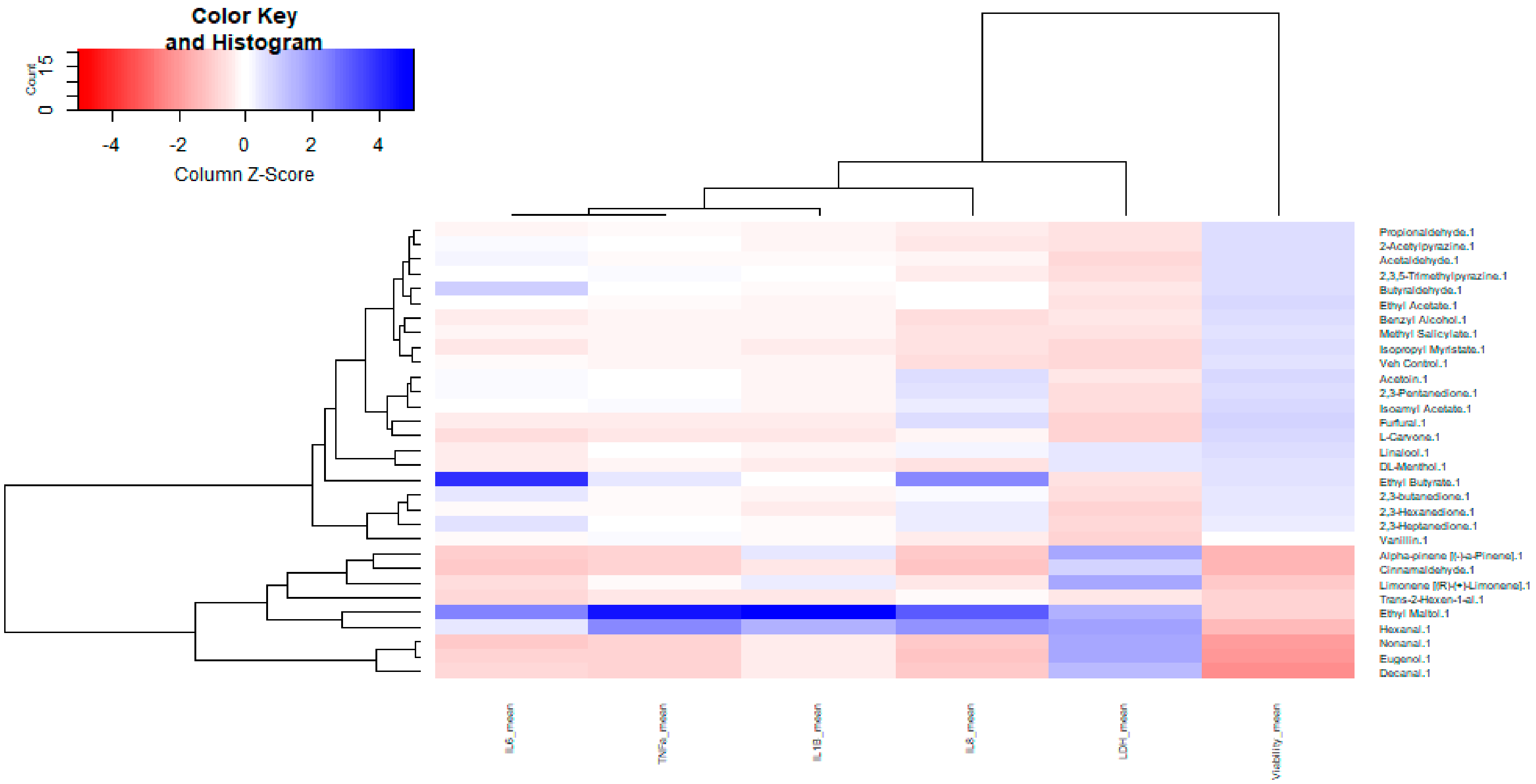


**Figure S9.** Heat map of clustering of toxicity tests in naïve THP-1. A) 4 hour B) 24 hour.

A)

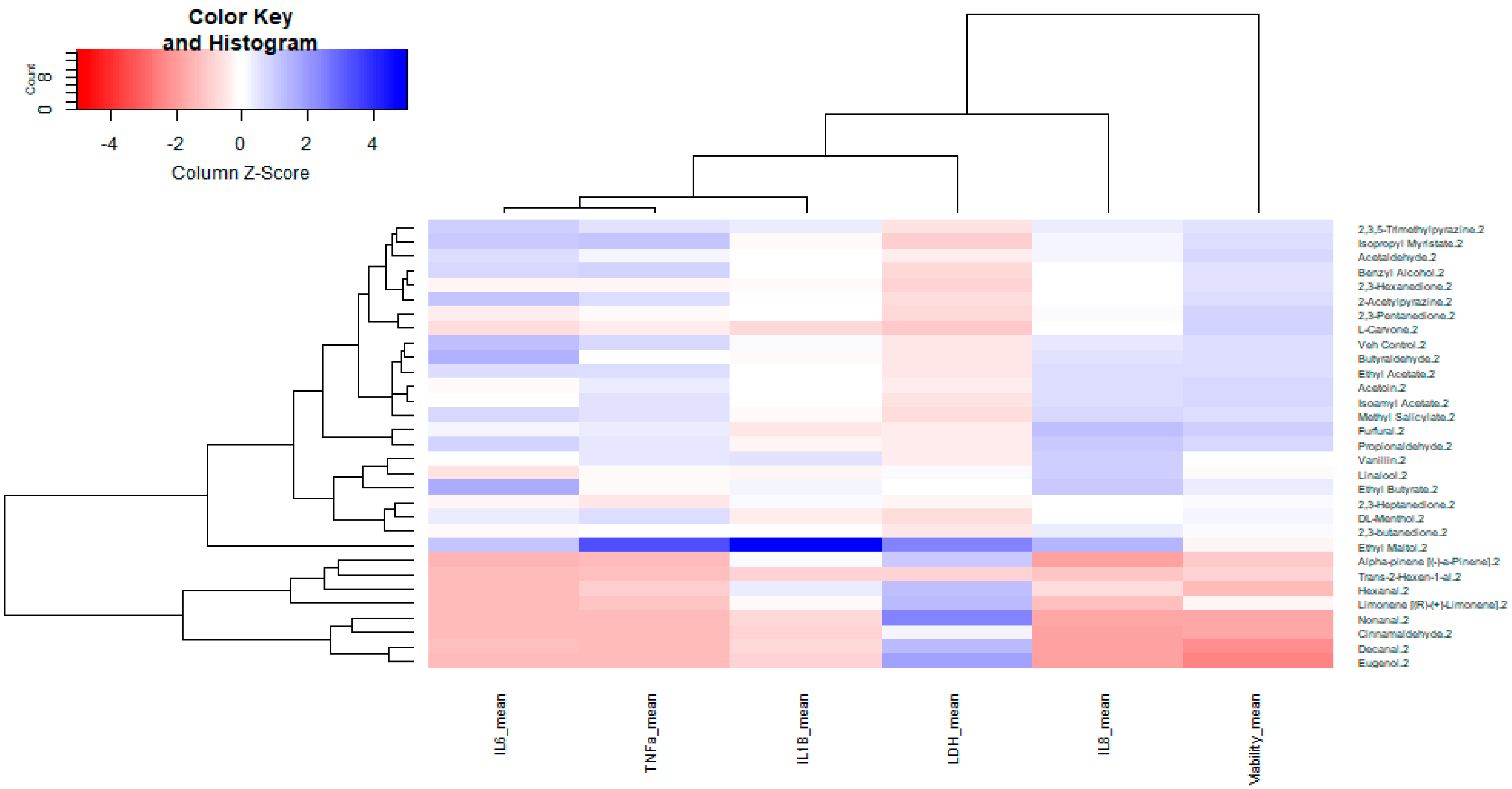
Color Key
and Histogram
Count
0
8
-4
-2
0
2
4
Column Z-Score
2,3,5-Trimethylpyrazine.2
Isopropyl Myristate.2
Acetaldehyde.2
Benzyl Alcohol.2
2,3-Hexanedione.2
2-Acetylpyrazine.2
2,3-Pentanedione.2
L-Carvone.2
Veh Control.2
Butyraldehyde.2
Ethyl Acetate.2
Acetoin.2
Isoamyl Acetate.2
Methyl Salicylate.2
Furfural.2
Propionaldehyde.2
Vanillin.2
Linalool.2
Ethyl Butyrate.2
2,3-Heptanedione.2
DL-Menthol.2
2,3-butanedione.2
Ethyl Maltol.2
Alpha-pinene [(-)-a-Pinene].2
Trans-2-Hexen-1-al.2
Hexanal.2
Limonene [(R)-(+)-Limonene].2
Nonanal.2
Cinnamaldehyde.2
Decanal.2
Eugenol.2
IL6_mean
TNFa_mean
IL1B_mean
LDH_mean
IL8_mean
Viability_mean

B)

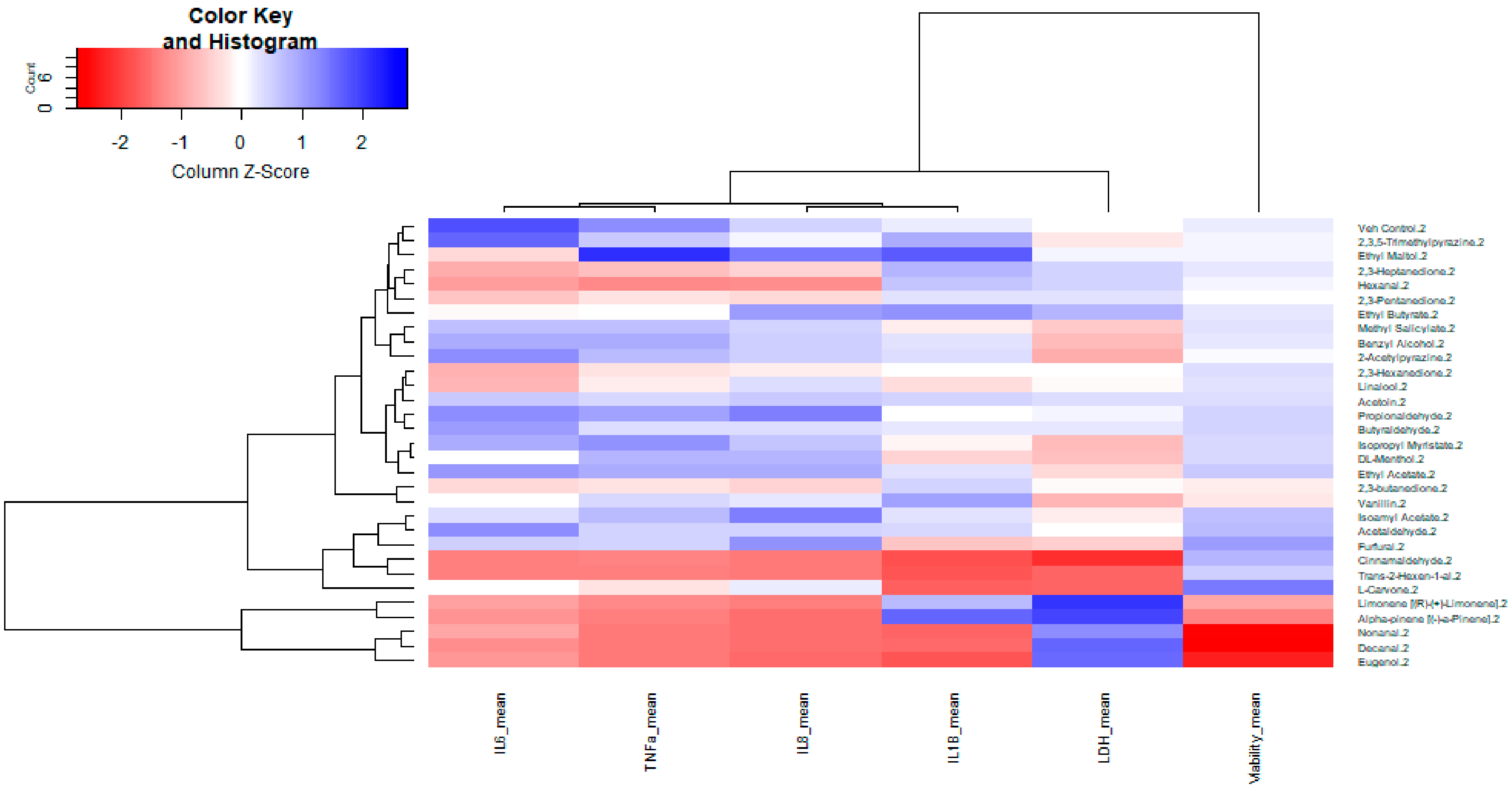


**Figure S10.** Heat map of clustering of toxicity tests in activated THP-1. A) 4 hour B) 24 hour.

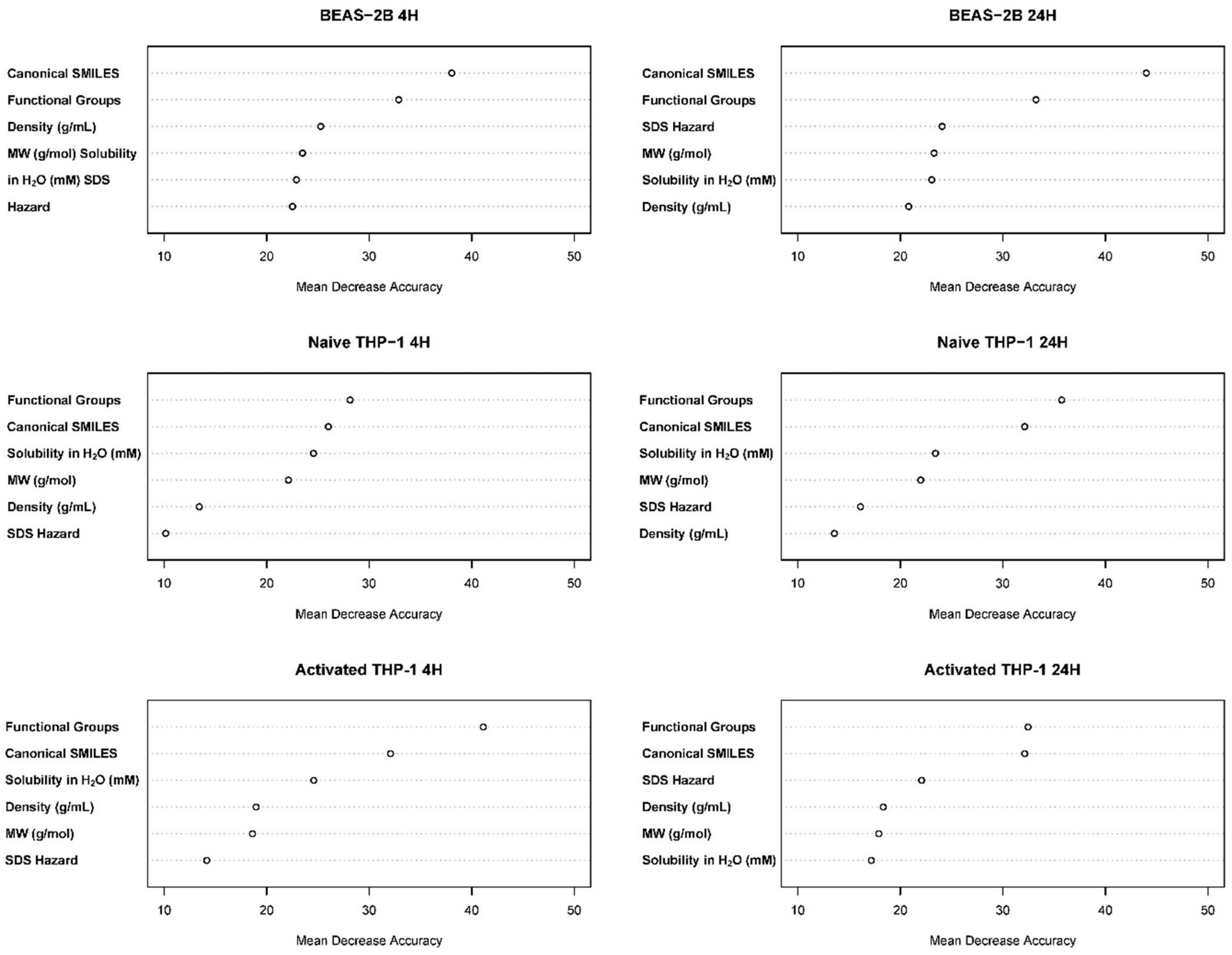


**Figure S11.** Important physicochemical properties in accurately predicting a compound's toxicity.

## References


34. Färber, I., Günnemann, S., Kriegel, H., Kröger, P., Müller, E., Schubert, E., Seidl, T., Zimek, A., Muenchen, L., 2010. On Using Class-Labels in Evaluation of Clusterings. Proc. 1st International Workshop on Discovering, Summarizing and Using Multiple Clusterings (MultiClust 2010). https://www.researchgate.net/publication/228374158_On_using_class-labels_in_evaluation_of_clusterings [site accessed Sep 21 2020].
35. Kriegel, H., Kröger, P., Sander, J., Zimek, A., 2011. "Density-based Clustering". WIREs Data Mining and Knowledge Discovery, 1(3): 231–240. doi:10.1002/widm.30.
56. Liaw, A., Wiener, M., 2002. Classification and regression by random forest. R News 2(3), 18--22. https://cogns.northwestern.edu/cbmg/LiawAndWiener2002.pdf [site accessed 09/20/20].
57. R Core Team, 2019. R: A language and environment for statistical computing. R Foundation for Statistical Computing, Vienna, Austria. URL https://www.R-project.org/. [site accessed 09/20/20].
